# Heterogeneity-Driven Strengthening and Hardening in Heterostructured Materials: Modeling and Simulation Across Length Scales

**DOI:** 10.3390/ma19112334

**Published:** 2026-06-01

**Authors:** Caizhi Zhou, Md Mahabubur Rohoman, Nan Li

**Affiliations:** 1 Department of Mechanical Engineering, University of South Carolina, Columbia, SC 29208, USA; 2 Center for Integrated Nanotechnologies, Los Alamos National Laboratory, Los Alamos, NM 87545, USA

**Keywords:** heterostructured materials, hetero-deformation-induced hardening, geometrically necessary dislocations, strain gradient plasticity, crystal plasticity modeling, dislocation dynamics simulation, molecular dynamics simulation

## Abstract

Heterostructured metals and alloys are designed with spatial variations in strength and hardening that produce synergy beyond the rule of mixtures. This review surveys face-centered cubic (FCC), body-centered cubic (BCC), and hexagonal close-packed (HCP) systems, including architectures formed or modified by rolling and related severe plastic deformation routes, and examines them under tension, compression, and shear. Across material classes, mechanical incompatibility between hetero-zones drives stress partitioning and plastic strain gradients that store geometrically necessary dislocations near zone boundaries. The associated internal back and forward stresses sustain work hardening, delay instability, and influence localization and damage initiation. We evaluate continuum, crystal plasticity, dislocation-based mesoscale, and atomistic approaches by whether they predict these internal fields and whether they are validated against internal-field measurements. Key observations are that predictive models require physically identifiable intrinsic length scales, experimentally constrained interface laws, and careful separation of mechanisms to avoid double-counting when gradient and kinematic terms coexist. Major gaps remain in parameter identifiability for multi-zone and nonlocal formulations, in transferability across processing routes and loading modes, and in community benchmarks that couple well-characterized microstructures with multimodal measurements. Recommendations are provided for validation targets and benchmark campaigns to accelerate predictive design.

## 1. Introduction

**Figure 1:**
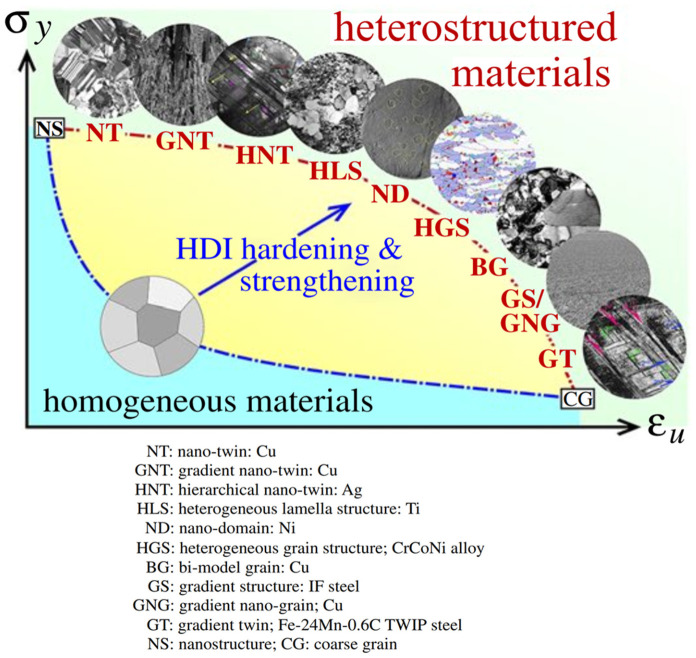
Property–space overview for gradient and lamellar heterostructures, showing that deliberate spatial variation in strength and length scale can produce simultaneous gains yield strength (σ_y_) and uniform elongation (ε_u_) [4].

Heterostructured materials are engineered solids in which the microstructure, and consequently the local constitutive response, varies systematically in space. The underlying objective is to achieve heterogeneity-driven strengthening and hardening, wherein mechanical incompatibility among hetero-zones generates internal constraints, long-range internal stresses, and sustained strain hardening. Based on this principle, a materials perspective was developed in which heterogeneous architectures are recognized as enabling property combinations that surpass predictions based on simple averaging bases [1]. Subsequent work further highlighted the concept of hetero-deformation-induced (HDI) hardening and its mechanistic association with the development of back and forward stresses [2]. Extensive studies have examined design strategies for heterogeneous nanostructures, emphasizing the emergence of strength–ductility synergy beyond conventional rule-of-mixtures expectations [3]. These foundational concepts have since been extended to a broad range of material systems, including gradient-structured [4], lamellar [5], and high-entropy alloys [6,7]. As summarized in Figure 1, gradient and lamellar heterostructures can access combinations of strength and tensile ductility that are difficult to achieve in homogeneous alloys.

**Figure 2:**
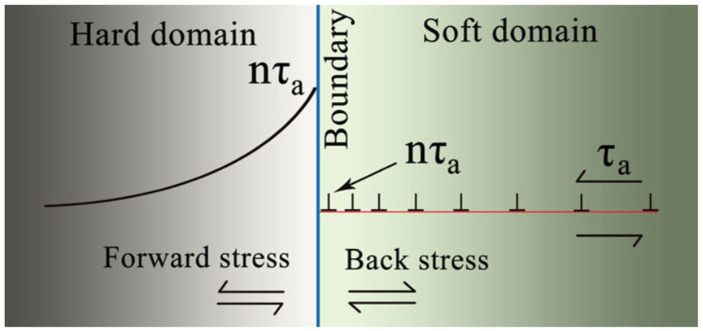
Schematic of GND pile-up at a hetero-zone boundary and the resulting back/forward stress partition that underpins HDI hardening [2]. τ_a_ represents the applied shear stress.

Experimental studies have demonstrated that gradient-structured metals can exhibit exceptional work-hardening capacity and enhanced uniform elongation in both face-centered cubic (fcc) and body-centered cubic (bcc) systems [8,9,10,11]. Furthermore, heterogeneous lamellar and heterogeneous grain architectures have been shown to combine the high strength characteristic of ultrafine-grained regions with the ductility typically associated with coarse-grained domains [12,13,14]. In multilayer laminates and dissimilar-metal laminates, interface-affected zones and gradient transition layers contribute additional strengthening while preserving stable plastic flow [15,16,17,18,19,20,21,22]. Harmonic and core–shell structures produced via powder-processing routes provide a complementary network heterogeneity that mechanically constrains softer regions while maintaining continuous ductile load paths [23,24,25]. Across these systems, incompatibility is the central physical thread. When adjacent zones differ in yield strength or hardening capacity, equilibrium and compatibility enforce stress partitioning and plastic strain gradients that evolve with deformation. These gradients require storage of geometrically necessary dislocations (GNDs) near hetero-zone boundaries, which produces back stress in softer regions and forward stress in harder regions. This coupling is commonly framed as HDI strengthening and hardening [2,9,26,27]. Stress partitioning has been quantified directly in multilayer steels using in situ neutron diffraction [22], and three-dimensional X-ray microdiffraction has resolved submicron-scale mechanical heterogeneity at grain scale [28]. In situ microscopy at heterostructured interfaces further revealed dislocation pile-ups and transmission processes that underpin many interface laws used in simulations [29]. These datasets provide direct constraints for models. The diversity of such architectural strategies motivates a review that systematically compares classes of modeling approaches based on common physical objectives and experimentally accessible observables, rather than treating each material system as an isolated case. The extra work hardening in heterostructured materials arises from plastic incompatibility between neighboring hetero-zones, which generates GND accumulation and internal back/forward stresses. Figure 2 schematically illustrates this HDI-hardening mechanism and therefore provides the physical basis for the discussions that follow.

Modeling and simulation are essential because the decisive quantities are internal fields that cannot be inferred from a macroscopic stress–strain curve. Continuum constitutive models, including multi-zone frameworks and strain-gradient plasticity, can capture heterogeneity effects efficiently when their length scales are identifiable [30,31,32]. Crystal plasticity finite element and spectral approaches resolve slip, twinning, and texture effects and can predict full-field strain and lattice strain maps for direct comparison to experiments [33,34,35]. Mesoscale dislocation-based methods provide mechanistic scaling relations for pile-ups and interface transmission [36,37], while atomistics quantify dislocation-interface interactions and interface strength at the nanoscale [38,39]. This review compares these modeling classes using common targets and observables.

Modeling and simulation are essential in the study of heterostructured materials because the governing quantities are internal field variables that cannot be reliably inferred from macroscopic stress–strain responses alone. Continuum constitutive models, including multi-zone frameworks and strain-gradient plasticity theories, can efficiently represent heterogeneity-induced effects provided that their intrinsic length scales are physically identifiable and experimentally constrained [30,31,32]. Crystal plasticity models, implemented through finite element or spectral methods, explicitly resolve slip, twinning, and texture evolution, and can predict full-field strain and lattice-strain distributions for direct comparison with experimental measurements [33,34,35]. Mesoscale dislocation-based models establish mechanistic scaling relationships for dislocation pile-ups and transmission across interfaces [36,37], whereas atomistic simulations quantify dislocation-interface interactions and interfacial strength at nanometer length scales [38,39]. The present review evaluates these modeling classes using shared physical targets and experimentally accessible observables.

Recent review and perspective articles [40,41,42,43,44,45,46,47] offer complementary entry points into heterostructured materials, and they may be consulted selectively. The hetero-zone interaction concept and a design perspective for HDI stresses were established in [40,41]. Gradient nanostructured metals are reviewed with emphasis on processing, property trends, and deformation mechanisms in [42]. Harmonic structures, broader classes of heterogeneous metals, and application-oriented surveys are covered in [43,44,45]. Conceptual paradoxes in applying classical strengthening laws, general routes to strength–ductility synergy, and heterostructured stainless steels are discussed in [46,47]. In contrast, the present review centers on multiscale modeling and simulation, with validation-focused treatment of stress partitioning, GND storage, flow stability, and damage evolution.

The article is organized beginning with deformation physics and digital microstructure representation, followed by modeling strategies across length scales, and concluding with discussions of damage, calibration methodologies, and emerging research directions. Each modeling approach is assessed using a common evaluative framework. This framework examines the required microstructural inputs and characteristic length scales, the physical mechanisms represented explicitly, the experimental observables employed for validation, and the numerical or conceptual failure modes most frequently encountered. This structured approach is intended to guide informed model selection and to clarify the experimental developments necessary for rigorous and decisive validation.

## 2. Taxonomy of Heterostructured Architectures and Microstructural Features

**Figure 3:**
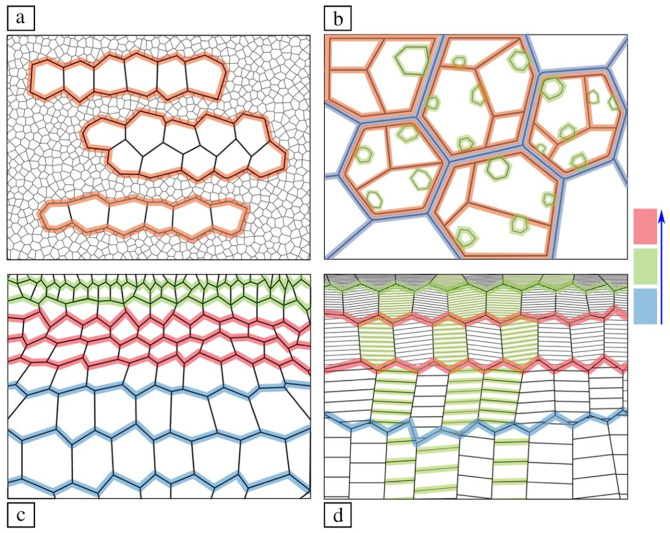
Representative strain-gradient architectures in heterostructured grains [4]. (**a**) Heterogeneous lamellar structure. (**b**) Heterogeneous grain structure. (**c**) Gradient structure. (**d**) Gradient nano-twinned structure. Color scale bar: qualitative comparison of strain gradient. The orange color highlights regions with higher strain gradients. The blue arrow indicate the direction of the strain gradient from low-gradient to high-gradient regions.

Heterostructured architectures may be classified according to the spatial arrangement of hetero-zones and the dominant contrast governing their mechanical response. Here, a hetero-zone denotes a spatial region with an approximately uniform microstructural state and constitutive response, such as a hard surface layer, a ductile core, or a phase layer within a laminate. Hetero-zone boundaries include sharp interfaces and graded transition layers, where incompatibility-driven GND storage and slip transfer govern inter-zone coupling. Layered laminates and heterogeneous lamellar configurations are among the most extensively studied systems, offering well-defined geometries that facilitate analysis of stress partitioning [12,17,18]. Gradient structures, frequently produced through severe plastic deformation, exhibit continuous spatial variations in microstructure that promote back-stress strengthening mechanisms [8,10,11]. Bimodal grain size distributions, characterized by coarse-grained domains embedded within an ultrafine-grained matrix, provide an alternative strategy for strain delocalization [48,49,50]. Harmonic or core–shell architectures generate percolating hard shells surrounding ductile cores, thereby enhancing kinematic constraint effects [23,24,25]. Multiphase heterostructures introduce additional phase-boundary mechanisms that govern load transfer and contribute to hardening behavior [51,52,53]. Hierarchical designs integrate these concepts across multiple length scales, as exemplified by multilevel steels and entropy alloys [53,54,55,56], as well as gradient nanolaminate medium-entropy alloys fabricated via shape-preserving machining [57]. This taxonomy provides a useful framework for identifying the specific constraints and characteristic length scales that a given modeling approach must resolve. Figure 3 provides a compact visual taxonomy of representative gradient and lamellar architectures discussed in this section. Gradient-structured materials are characterized by a systematic variation in a microstructural parameter along a single spatial direction [58], most commonly grain size, dislocation density, or twin density. In practice, this microstructural parameter denotes a measurable state variable such as grain size, twin spacing, or defect density, and the gradient is characterized by its depth-dependent profile and a characteristic gradient thickness or length scale. These geometric attributes control the strength contrast among layers and set the heterogeneity length scale that governs incompatibility stresses and the relevance of gradient-sensitive constitutive terms.

Gradient-structured materials are characterized by a systematic variation in a microstructural parameter along a single spatial direction [58], most commonly grain size, dislocation density, or twin density. Experimental investigations in copper and steels have demonstrated that such gradients can enhance yield strength while sustaining strain hardening through back-stress strengthening mechanisms [8,9,10]. Gradient nanotwinned metals and gradient-nanotwinned steels introduce additional twin-boundary length scales that further influence mechanical response [54,59,60,61]. Moreover, superimposed gradients in residual stress or crystallographic texture have been shown to affect cyclic behavior and tension-compression asymmetry [62,63,64]. Both physics-based models and microstructure-resolved simulations have been developed to describe deformation in gradient nanostructured metals [65,66,67,68]. Representative benchmark systems include gradient nanograined copper [69,70], cryogenically processed gradient cell-structured alloys [71], and gradient-confined metallic glasses.

Layered and laminate heterostructures, in contrast, impose sharp or graded strength transitions between adjacent layers. Strong interfacial bonding can stabilize plastic flow and delay the onset of necking in both coarse-grained and nanostructured laminates [12,17,72,73]. In dissimilar-metal laminates, interface-affected zones frequently govern both strength and ductility [15,18]. Graded transition layers can mitigate stress concentrations while preserving substantial strain gradients that promote HDI hardening [16,74,75]. In age-strengthened aluminum alloy laminates, the coupled effects of precipitation and mechanical incompatibility during deformation can generate additional strengthening and hardening [76]. In heterogeneous lamellar structures, heterogeneity is distributed at the colony scale and often promotes dispersed strain banding.

**Figure 4:**
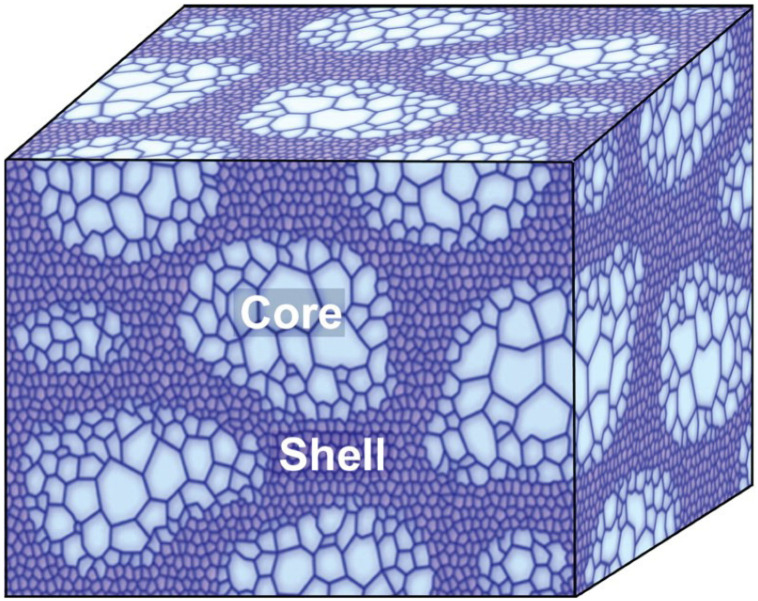
Schematic of harmonic-structure design in powder-processed Ti, showing hard shell regions surrounding softer cores and the resulting three-dimensional shell connectivity [80].

Bimodal and multimodal grain size distributions constitute forms of heterogeneity that do not possess a single dominant spatial direction. Critical, controllable parameters include the volume fraction of coarse grains, the size ratio between grain populations, and the spatial connectivity of the coarse-grained fraction [49,77,78]. Systematic investigations in bimodal nickel have demonstrated how variations in size ratio and coarse-grain fraction influence strain partitioning behavior [79]. In harmonic or core–shell architectures, powder-based processing generates a network of fine-grained shell regions that percolate around coarser cores. Both experimental observations and modeling analyses indicate that back-stress development and strain partitioning are strongly dependent on whether the fine-grained shell forms a continuous load-bearing skeleton [23,24,80]. Harmonic structures as shown in Figure 4 are topologically distinct from simple one-dimensional gradients because a connected hard shell network constrains the softer cores and alters load transfer. Three-dimensional heterogeneous nanostructures further extend this concept by employing interlocking architectures to regulate connectivity and load-transfer pathways [81]. Such structural classes challenge simplified two-zone mixture models, as mechanical constraint is governed predominantly by topology and percolation rather than solely by phase fractions.

The microstructural information available for modeling is intrinsically linked to the measurement modality employed. Electron backscatter diffraction (EBSD) mapping provides grain size distributions, crystallographic textures, and curvature-based estimates of GND density, whereas diffraction and microdiffraction techniques enable quantification of load sharing and internal stress partitioning [22,28]. For layered or composite architectures, three-dimensional interface topology, which is critical for crack propagation paths, can be characterized through tomography and serial sectioning [82,83]. Given the inherent uncertainties associated with such measurements, simulation strategies often benefit from ensembles of statistically equivalent microstructures rather than reliance on a single deterministic reconstruction. Subsequent sections relate these experimental characterization approaches to the digital representations employed in continuum, crystal plasticity, and mesoscale modeling frameworks.

## 3. Key Deformation Physics in Heterostructured Materials

Models of deformation in heterostructured materials must capture a tightly coupled set of physical phenomena that arise from mechanical incompatibility among hetero-zones. These phenomena establish the linkage between microstructural heterogeneity, the evolution of internal field variables, and the resulting macroscopic responses, including strengthening, strain hardening, and failure. The discussion that follows organizes these requirements into two principal clusters that recur across diverse architectural classes and modeling scales.

### 3.1. Incompatibility, Stress Partitioning, and HDI Strengthening and Hardening

**Figure 5:**
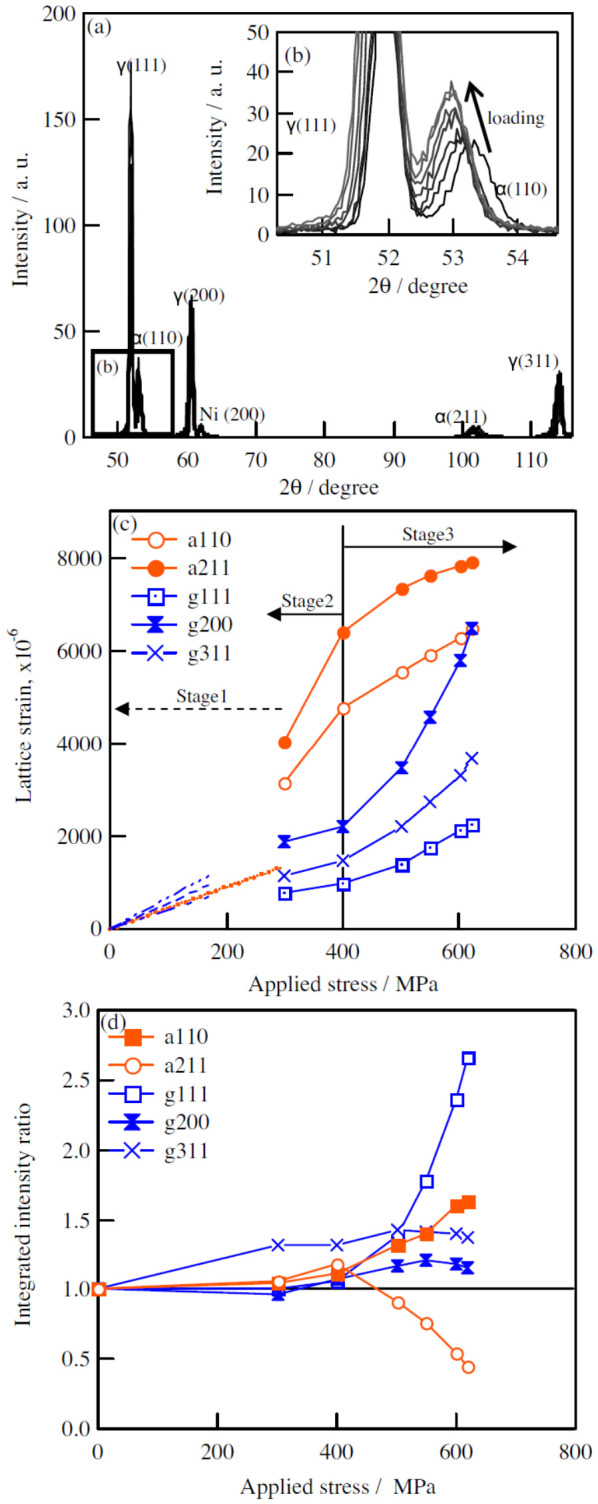
In situ neutron-diffraction evidence of stress partitioning through lattice-strain evolution in multilayer steels [22]. (**a**) Whole profiles obtained by in situ neutron diffraction measurements. (**b**) Close-up view of 111 in fcc phase and 200 in bcc phase. (**c**) Lattice strain-applied stress responses in multilayered steels consisting of as-quenched martensite (a110 and a211) and austenite (g111, g200 and g311). (**d**) Integrated peak intensities from martensitic and austenitic phase of multilayered steel as a function of applied stress normalized by the initial intensity obtained from profile before deformation.

Mechanical incompatibility between adjacent hetero-zones constitutes the fundamental origin of the enhanced strengthening and strain hardening observed in heterostructured materials. When neighboring zones differ in yield strength or hardening behavior, mechanical equilibrium requires continuity of traction across their interfaces, while kinematic compatibility constrains relative displacement. The coupled enforcement of these conditions gives rise to strain-dependent stress partitioning between zones. Ojima et al. quantified layer-resolved load sharing in multilayer steels by in situ neutron diffraction [22]. As shown in Figure 5, in situ neutron diffraction can resolve layer-by-layer lattice-strain evolution and thus directly quantify stress partitioning in multilayer steels. Similar trends have been captured by zone-coupled laminate and gradient models that impose equilibrium and compatibility explicitly [31,84] and provide benchmarks for verification.

Stress partitioning evolves with increasing strain as a consequence of differences in hardening rates among hetero-zones [2,9]. When a mechanically softer zone exhibits rapid strain hardening, the stress contrast between adjacent regions may diminish, promoting a more homogeneous deformation field. Conversely, limited hardening in one region can intensify stress partitioning and accelerate strain localization. The loading mode further influences these interactions. Under cyclic loading, kinematic effects and the evolution of residual stresses modify stress partitioning during each half-cycle. Experimental studies have demonstrated that residual stress fields can contribute significantly to tension–compression asymmetry in gradient-structured materials [63,64]. Accurate reproduction of these behaviors requires constitutive formulations capable of representing both the initial strength contrast and the differential hardening responses of the constituent zones.

**Figure 6:**
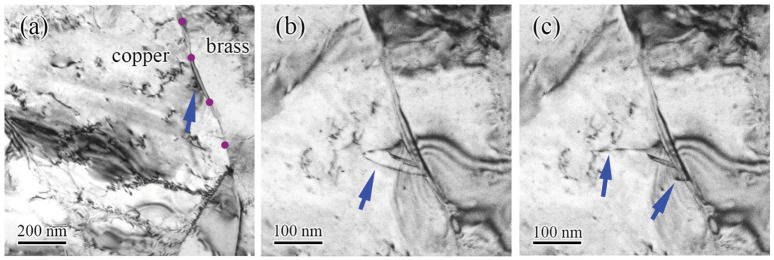
Direct in situ view of dislocation emission and interaction at a Cu/brass heterointerface [29]. (**a**) A Cu grain-brass interface marked by four purple dots. (**b**) A dislocation emitted from the interface. (**c**) The dislocation reached the sample surface and broke into two segments. (The blue arrows mark the locations of stress concentration).

Stress partitioning is accompanied by plastic strain gradients that concentrate near boundaries and transition layers. For example, in a two-layer laminate under tension, the softer layer yields first and accumulates a larger plastic strain while the harder layer remains less deformed. Displacement continuity then requires the plastic strain to transition from one layer value to the other across a finite interfacial region, which produces a localized plastic strain gradient. The associated GND storage generates long-range internal stresses that feed back into macroscopic hardening. In crystalline metals, lattice curvature requires the storage of GNDs, and Nye kinematics link GND measures to gradients of plastic distortion [85]. Zhou et al. observed interface-proximal dislocation activity in situ in heterostructured interfaces, including pile-ups and transmission events (Figure 6) that generate long-range internal stresses [29]. These internal stresses appear as back stress in softer zones and forward stress in harder zones [2,26]. This partition of these internal stresses provides a mechanistic basis for HDI strengthening and for the high strain hardening observed in many heterostructures [2,9].

HDI hardening does not exhibit a linear dependence on the nominal strain gradient. Investigations of copper–brass laminates have demonstrated that HDI hardening can saturate as dislocation structures reorganize in the vicinity of interfaces [86]. This finding indicates that kinematic hardening formulations should incorporate physically motivated saturation mechanisms associated with the evolution of dislocation substructures, rather than relying solely on numerical regularization or damping parameters. Architectural design can further modify the balance between GND accumulation and dynamic recovery. For example, the combination of a gradient structure with transformation-induced plasticity (TRIP) in austenitic stainless steel has been shown to sustain hardening through the activation of additional deformation mechanisms with increasing depth [87]. Similarly, gradient hierarchical nanotwinned structures introduce supplementary dislocation storage sites and alter the evolution of internal stress fields during continued deformation [54].

Across these material systems, predictive modeling requires accurate representation of the coupled evolution of stress partitioning, strain-gradient-driven GND storage, and the resulting internal stresses. Accordingly, validation protocols should incorporate at least one internal-field observable, such as diffraction-based stress partitioning maps [22] or microdiffraction and microscopy evidence of interface-localized dislocation processes [28,29], rather than relying exclusively on macroscopic stress–strain agreement obtained under a single loading configuration.

### 3.2. Flow Stability, Strain Localization, and Damage Precursors

**Figure 7:**
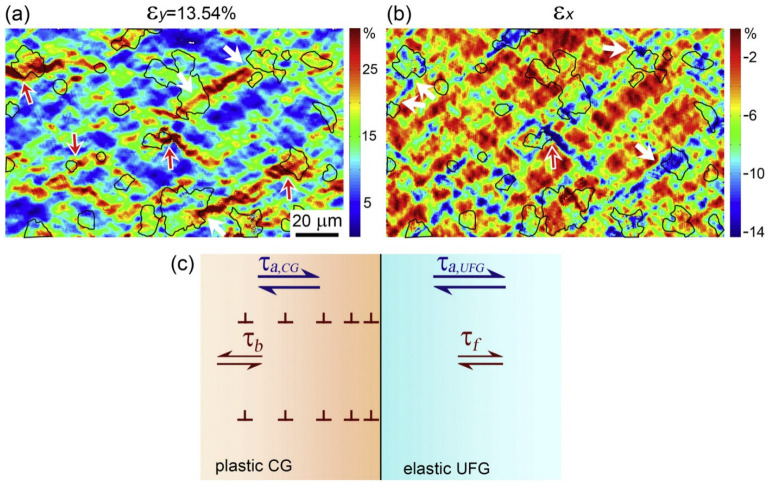
High-resolution strain maps showing the initiation, propagation, and interaction of dispersive shear bands in heterostructured Cu [72]. (**a**) *ε*_y_ strain map and (**b**) *ε*_x_ strain map. The solid lines outlined the boundary of coarse-grained domains. (**c**) Schematic illustration of the dislocation pile-up-induced long-range back stress (τ_b_) and forward stress (τ_f_) around domain boundary. This figure demonstrates that heterogeneity can spatially distribute plastic localization, thereby delaying the formation of a single catastrophic band and improving flow stability. (The white arrows indicate the arresting and buffering effects of the coarse-grained (CG) domains on shear-band propagation, while the red arrows highlight regions of elevated strain concentration near the domain boundaries. The black solid lines delineate the boundaries of the CG domains).

Many heterostructured materials achieve enhanced ductility not by reducing strength, but by promoting the spatially distributed plastic deformation. In this context, spatially distributed indicates that plastic strain is carried by multiple bands and domains across the specimen rather than concentrating into a single dominant shear band. In gradient architectures, shear bands that would localize into narrow regions can extend across the gradient, thereby becoming delocalized and postponing catastrophic failure [88]. In heterostructured copper, dispersed shear bands and microshear bands accommodate mechanical incompatibility while preserving substantial strain hardening [72,73], as illustrated in Figure 7. Enhanced work hardening in gradient microstructures has further been associated with hierarchical strain-band formation and the progressive activation of new bands across distinct zones [89,90]. Accurate representation of these phenomena requires modeling frameworks capable of predicting strain-band statistics and their evolution, rather than reproducing only average hardening behavior.

Conversely, heterogeneity may exacerbate harmful localization when strength contrast is excessive or transition layers are insufficiently graded. In dissimilar-metal laminates, interfacial shear and delamination may arise when the interface is weaker than adjacent regions or when mechanical constraint generates elevated stress triaxiality near the interface [21,91]. Under high strain-rate loading, adiabatic shear localization and thermally induced softening can become dominant, with their interaction with heterogeneity governed in part by stacking-fault energy and strain-rate sensitivity [92,93]. Predictive modeling under such conditions therefore necessitates thermomechanical coupling when the loading regime renders it significant.

Localization phenomena in heterostructured materials are governed by the competition between strain hardening within mechanically constrained regions and softening due to damage evolution or thermal effects. Stability analyses and numerical bifurcation studies have established quantitative relationships between intrinsic gradient length scales and critical shear-band spacing, thereby clarifying the conditions under which dispersed banding transitions to a dominant, localized shear band [94,95,96,97]. In layered and dual-phase systems, interface character and topology determine whether localization initiates in the soft zone, at the interface, or in the hard zone. Numerical simulations further demonstrate that modest variations in mechanical constraint can invert the predicted initiation site [98,99,100,101]. Given the inherently stochastic nature of localization at the microstructural scale, predictive models intended to assess ductility or failure should report statistical measures, such as localization correlation lengths and strain-band spacing distributions, rather than relying solely on isolated field snapshots. These statistical targets can be extracted consistently from high-resolution digital image correlation (HR-DIC) data and simulated strain fields, thereby enabling direct and quantitative model validation.

The experimental observables emphasized throughout this review include diffraction-derived stress partitioning maps, full-field strain distributions and strain-band statistics obtained from DIC, and microstructure-informed proxies for GND density derived from EBSD curvature and related analyses [22,28]. In fracture-focused investigations, crack-path topology and its correlation with architectural features and local microstructural characteristics constitute additional validation targets [102,103]. Established modeling drawbacks include boundary-condition artifacts that can dominate apparent stress partitioning in small representative volume elements, as well as mesh-dependent localization that may be misinterpreted as physically meaningful band formation in softening formulations [35]. Accordingly, credible modeling efforts require rigorous verification, systematic sensitivity analyses, and transparent reporting of predicted internal fields together with associated uncertainties.

In summary, while the physical phenomena of incompatibility-driven stress partitioning, GND-mediated back stresses, and strain delocalization are well established, the literature remains disproportionately focused on monotonic, low-rate loading of idealized planar architectures. Critical gaps persist in the quantitative understanding of how these mechanisms couple under multiaxial, cyclic, or high strain-rate conditions, particularly in three-dimensional topologies such as harmonic or hierarchical structures. Furthermore, most experimental validations report either stress partitioning or strain localization, but rarely both from the same specimen under identical loading, limiting the constraints available for model calibration. The field would benefit from systematic studies that simultaneously quantify the evolution of internal stresses, GND densities, and localization patterns across multiple architectures, thereby exposing which physical couplings are genuinely predictive and which remain case-dependent fitting constructs.

## 4. Digital Representation of Heterostructures for Simulation

The predictive capability of simulations for heterostructured materials depends on the reliability with which microstructural heterogeneity is represented in digital form. Digital microstructures must encode the essential architectural features, such as spatial arrangement, property contrast, geometric configuration, and transition characteristics, that govern deformation behavior, while remaining consistent with the imposed boundary conditions and the numerical constraints of the chosen solver. Two complementary strategies are prevalent in the literature. The first involves the generation of synthetic microstructures designed to reproduce prescribed statistical descriptors. The second relies on experimental reconstruction, wherein digital representations are derived directly from measured microstructural data.

### 4.1. Synthetic Microstructure Generation

Synthetic microstructure generation is particularly advantageous when the objective is parametric exploration of architectural variations or when high-fidelity experimental reconstructions are unavailable. Tessellation-based polycrystalline constructions and voxelized representations can be improved with prescribed spatial gradients in grain size, dislocation density, or twin density to produce digital gradient structures consistent with experimentally measured profiles [104,105]. Open-source pipelines, such as Dream.3D [104], support this workflow by generating statistically equivalent three-dimensional polycrystals from prescribed texture and grain-size statistics and by exporting voxelized or meshed representative volume elements (RVEs). In heterostructures, such tools are useful for constructing graded microstructures by prescribing spatially varying statistics and for enforcing connectivity constraints in core–shell or harmonic architectures. Yu used grain size distribution profiles to predict the overall response of gradient nanograined materials, which provides a practical route for defining synthetic gradients when only a target profile is available [104]. For bimodal and harmonic architectures as shown in Figure 4, Sawangrat et al. [23] and Park et al. [24] showed that the connectivity and percolation of mechanically hard and soft regions exert a dominant influence on load-transfer pathways and strain delocalization [81]. In dual-phase steels, representative volume element (RVE)-based optimization and response-surface methodologies have illustrated how synthetic RVEs can be calibrated to satisfy prescribed statistical descriptors while enabling systematic sensitivity analyses [106]. Moreover, synthetic microstructure ensembles provide a robust framework for uncertainty quantification, as they facilitate the propagation of variability in microstructural inputs into predictions of stress partitioning, strain localization, and related internal field responses.

A critical modeling decision in synthetic microstructure generation concerns the representation of transition layers and boundary-affected regions. Explicit transition layers may be incorporated by grading material properties over a finite thickness informed by measurements of interface-affected zones [15] and by conceptual frameworks describing hetero-zone boundary-affected regions [26,27]. When such transition layers are not resolved directly, equivalent physical effects must be represented through nonlocal or strain-gradient formulations. In these cases, the intrinsic length scales embedded in the constitutive model should be calibrated and explicitly compared with the characteristic length scale of the imposed heterogeneity to ensure physical consistency [30,107]. Accordingly, synthetic microstructures should be documented together with their target statistical descriptors and resolution metrics, enabling independent assessment of representativeness and reproducibility.

### 4.2. Experimental Reconstruction of Microstructures

**Figure 8:**
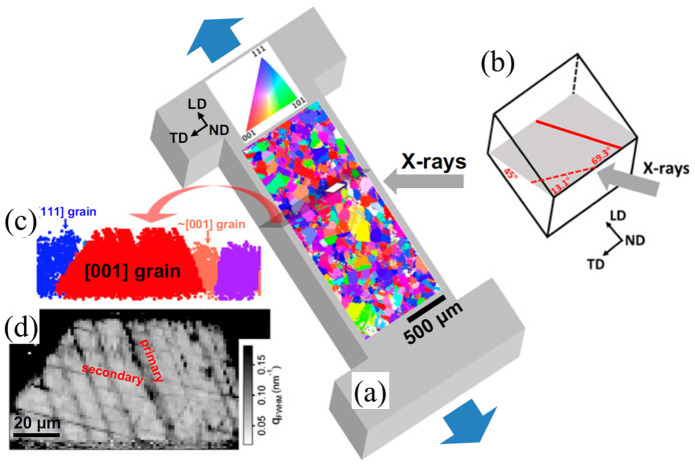
Experimental configuration and resulting orientation/strain mapping from three-dimensional X-ray microdiffraction [28]. (**a**) Schematic of a tensile specimen cut from a fatigued sample of stainless steel. The specimen is covered with a crystal orientation map of near-surface grains from μXRD measurement. (**b**) Schematic of an incident X-ray slicing plane (in gray) at 45° to the sample surface. (**c**) A crystal orientation map slicing along the X-ray beam direction. (**d**) A map of diffraction peak of the same [001] grain shown in (**c**). This figure demonstrates how reconstructed microstructure and associated lattice-strain fields can be extracted at submicron resolution for model input and validation.

Experimental reconstruction is generally preferred when the objective is quantitative validation against a specific material specimen. Two-dimensional EBSD characterization provides grain size distributions [82,83], crystallographic texture information, and curvature-based proxies for GND density. These datasets can be extended to three-dimensional representative volume elements (RVEs) through statistically informed reconstruction techniques. Diffraction and microdiffraction measurements furnish complementary internal-field information that may be directly mapped onto corresponding digital microstructures. Three-dimensional X-ray microdiffraction, for instance, can resolve submicron-scale heterogeneity, thereby constraining local constitutive parameter assignment [28]. In a typical measurement, a microfocused or microcollimated high-energy X-ray beam is raster-scanned across the specimen while diffraction patterns are collected at each position. Depth sensitivity can be obtained by differential-aperture or tomographic strategies, enabling three-dimensional maps of grain orientation and elastic lattice strain that directly quantify internal stress partitioning. Figure 8 is a representative example of experimentally reconstructed microstructure data that simultaneously provide structural and mechanical information. This kind of dataset is especially valuable because it supports both model parameterization and direct field-by-field validation. In situ diffraction experiments further enable quantification of zone-level load sharing in layered architectures [22]. For composite and laminate systems, tomography and serial sectioning are essential when three-dimensional interface topology governs crack propagation and damage evolution.

Experimental reconstruction inherently introduces uncertainties that should be treated explicitly in predictive simulations. EBSD-based reconstructions may fail to capture subsurface gradients and can underestimate three-dimensional connectivity, whereas tomographic methods are limited by finite spatial resolution and may smooth sharp transitions. When reconstructed microstructures are employed for predictive modeling, it is considered best practice to document the reconstruction methodology, spatial resolution, filtering or segmentation procedures, and the sensitivity of derived metrics, such as interface area density or transition-layer sharpness, to these choices. The use of ensemble-based reconstructions, which sample multiple statistically admissible three-dimensional configurations consistent with measured two-dimensional statistics, can mitigate reconstruction bias.

Boundary condition selection and loading control are particularly influential in heterostructure simulations because they govern the global constraint imposed on internal fields. Periodic boundary conditions are appropriate for bulk representative volume elements, whereas mixed or experimentally informed boundary conditions are required for configurations such as thin films, surface-gradient materials, and micropillar tests, where free surfaces and gripping constraints influence stress partitioning. Full-field crystal plasticity analyses of bimodal polycrystals have demonstrated that variations in boundary constraints can significantly alter predicted strain localization patterns, underscoring the need for explicit justification of boundary conditions in heterostructure modeling [35]. Representativeness further depends on the relationship between the size of the computational domain and characteristic heterogeneity and localization correlation lengths. Insufficiently large volume elements may bias localization behavior if they cannot accommodate multiple strain bands or interface segments. Accordingly, ensemble averaging and systematic size-scaling studies constitute essential components of credible digital representation practice.

The propagation of microstructural uncertainty into predictions of stress partitioning, strain localization, and failure metrics remains insufficiently reported. Practical approaches include sampling ensembles of digital microstructures, incorporating parameter distributions derived from calibration procedures, and reporting predictive intervals for field observables rather than only mean response curves [7,25]. Explicit uncertainty quantification is particularly critical when simulations are used to compare alternative heterostructure designs, as apparent performance rankings may change when variability is considered.

In summary, digital microstructure representation has advanced from idealized synthetic geometries toward statistically informed and experimentally reconstructed models. However, a critical weakness is the prevalent lack of uncertainty quantification: most simulations employ a single deterministic reconstruction or a narrowly sampled synthetic ensemble, ignoring specimen-to-specimen variability, measurement noise, and the inherent non-uniqueness of three-dimensional reconstructions from two-dimensional sections. The treatment of interfaces and transition layers remains particularly ad hoc, with few studies systematically comparing sharp-interface, graded-layer, and nonlocal formulations against the same experimental dataset. A key open question is whether the additional complexity of experimentally reconstructed microstructures consistently improves predictive accuracy for internal fields (e.g., GND distributions, localization hotspots) relative to simpler synthetic models that preserve only the essential statistical descriptors. Resolving this would provide rigorous justification for the substantial effort required to generate high-fidelity reconstructions.

## 5. Continuum-Scale Constitutive Modeling Strategies

**Figure 9:**
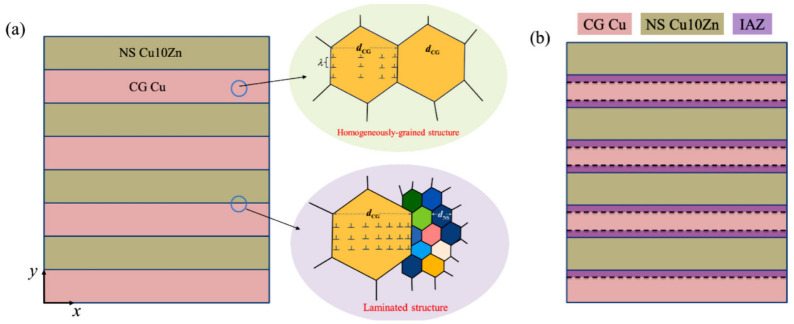
Constitutive idealization of a heterostructured laminate as a set of mechanically coupled layers or zones with incompatible plastic flow across interfaces [31]. (**a**) Schematic of dislocation pileups in homogeneously grained materials and in heterogeneous laminate. (**b**) The laminate considered as a multilayer composite consisting of three types of zones with distinct deformation mechanisms. This figure clarifies how architecture is reduced to a small number of interacting continua while retaining the essential physics of constraint and stress partitioning.

Continuum constitutive models continue to serve as primary tools for engineering prediction, owing to their compatibility with structural finite element implementations and their relative efficiency in calibration. However, for heterostructured materials, the principal requirement extends beyond reproducing zone-averaged behavior. Models must explicitly represent inter-regional coupling arising from mechanical incompatibility. Accordingly, many modeling frameworks adopt multi-zone formulations in which each hetero-zone is assigned a distinct flow rule and hardening law, while coupling among zones is enforced through equilibrium, kinematic compatibility, and interfacial constraints [31,108]. Figure 9 provides a clear bridge from physical heterostructure to continuum abstraction by representing the material as mechanically coupled layers with interfacial incompatibility. This figure clarifies how architecture is reduced to a small number of interacting continua while retaining the essential physics of constraint and stress partitioning. The same multi-zone abstraction can also represent stepwise gradients by discretizing a continuous gradient into multiple layers, and it can incorporate boundary-affected zones by assigning a finite-thickness interfacial region with distinct constitutive parameters. When calibrated against appropriate internal-field observables, such formulations can predict stress partitioning and provide actionable insights for heterostructure design. Due to space limitations, we do not provide a comprehensive review of the development of multi-zone plasticity methods. Section 5 is intended only to highlight their application in heterostructured materials.

### 5.1. Multi-Zone Plasticity Formulations

For various heterostructured architectures, continuum multi-zone plasticity formulations are grounded in small-strain $J_{2}$ flow theory [109]. Computational inelasticity frameworks formalize the additive decomposition of strain rate, $\dot{\varepsilon }={\dot{\varepsilon }}^{e}+{\dot{\varepsilon }}^{p}$, together with a consistent algorithmic structure that underpins numerous numerical implementations [110]. Within the small-strain framework, the total strain ε is additively decomposed into elastic and plastic parts, $\varepsilon =\varepsilon^{e}+\varepsilon^{p}$. The Cauchy stress is then related to the elastic strain by the linear elastic Hooke’s law, $\sigma =C:\varepsilon^{e}$, where C is the fourth-order stiffness tensor. This relation holds pointwise throughout the deformation history. However, solving for the stress and strain fields in a boundary value problem requires simultaneous satisfaction of equilibrium, compatibility, and the constitutive relations, typically achieved through incremental-iterative numerical methods (e.g., finite element analysis with a return-mapping algorithm). Thus, while the Hookean relation is locally valid, neither ε nor $\varepsilon^{e}$ is known a priori; they are determined as part of the global solution procedure. And yielding is commonly defined by a von Mises criterion [109]:

(1) $$f=\sigma_{\mathrm{eq}}-\sigma_{y}(\varepsilon_{\mathrm{eq}}^{p})=0$$

where the equivalent stress is given by $\sigma_{\mathrm{eq}}=\sqrt{\frac{3}{2}\;\sigma^{\prime }:\sigma^{\prime }}$ (deviatoric stress σ′) and evolving yield strength $\sigma_{y}$. The plastic strain rate follows the associative flow rule [109]:

(2) $${\dot{\varepsilon }}^{p}=\dot{\lambda }\;\frac{\partial f}{\partial \sigma }=\frac{3\dot{\lambda }}{2}\;\frac{\sigma^{\prime }}{\sigma_{\mathrm{eq}}}.$$

where $\dot{\lambda }$ is the plastic multiplier. Zones are coupled through equilibrium ∇⋅σ=0 and compatibility. For perfect bonding, displacements and tractions are continuous across interfaces: $u^{\left(1\right)}=u^{\left(2\right)}$ and $\sigma^{\left(1\right)}\cdot n=\sigma^{\left(2\right)}\cdot n$. These constraints constitute the minimal requirements for incompatibility-driven stress partitioning [31,108].

To capture long-range internal stresses from dislocation incompatibility, which is key to HDI hardening, a back stress tensor X is introduced. The yield surface shifts in stress space [111]:

(3) $$f=\left|\sigma -X\right|-\sigma_{y}=0.$$

Nonlinear kinematic hardening formulations introduce internal variables to represent back-stress evolution [111]. A commonly adopted evolution equation takes the form

(4) $$dX=\frac{2}{3}C\;d\varepsilon^{p}-\gamma X\;dp,$$

where C is the initial kinematic hardening modulus, γ controls saturation, and $dp=\sqrt{\frac{2}{3}d\varepsilon^{p}:d\varepsilon^{p}}$ is the accumulated plastic strain increment. In heterostructures, the HDI framework links X directly to GND density [9]. More general internal-variable frameworks have been developed to accommodate complex cyclic histories, an important consideration when heterostructures are evaluated under fatigue or load reversal [112].

To account for size effects and strain-gradient phenomena, phenomenological strain-gradient plasticity theories introduce an intrinsic material length scale l into the flow stress. As the flow stress depends on both plastic strain $\varepsilon^{p}$ and its gradient $\nabla \varepsilon^{p}$, Fleck and Hutchinson [113] formulated a higher-order theory where an effective strain E and effective strain gradient χ appear:

(5) $$\sigma_{\mathrm{flow}}=\sigma_{y}\;f\left(E,l\chi \right)$$

where $f\left(E,l\chi \right)=\sqrt{E^{2}+l^{2}\chi^{2}}$, thereby enabling regularization of localization while capturing gradient-induced strengthening in microstructurally graded materials [30,32,114].

Within continuum modeling of heterostructures, several fundamental issues arise. It is necessary to assess whether the chosen set of internal variables uniquely and adequately represents inter-zone coupling; how intrinsic length scales can be identified without overfitting; and how to prevent double-counting of physical mechanisms when both kinematic hardening and gradient terms are incorporated into the same formulation.

### 5.2. Multi-Zone Models for Heterostructured Materials

Multi-zone models are most transparent when the architecture admits a clear zone decomposition, as in planar laminates or stepwise gradients. Haouala et al. used computational homogenization to analyze how grain size influences strength in FCC polycrystals, illustrating how zone-based representations can be embedded within homogenization workflows [115]. Belgasam and Zbib demonstrated how RVE-based parametric studies can map microstructural choices to macroscopic response in dual-phase steels [107]. In these cases, extra strengthening can emerge naturally from constrained deformation even when each zone uses a conventional constitutive law. For gradient materials, zone-based discretizations approximate a continuous profile and can reproduce optimal partitioning patterns predicted in numerical studies [116]. For dual-phase or particulate systems, micromechanical homogenization provides a complementary route where local fields emerge from the distribution of properties and geometry.

Multi-zone formulations are particularly transparent and effective when the material architecture enables a well-defined decomposition into discrete regions, as in planar laminates or stepwise gradient structures. Computational homogenization approaches have been employed to examine the influence of grain size on strength in FCC polycrystals, demonstrating how zone-based representations can be systematically embedded within multiscale homogenization frameworks [115]. Similarly, Belgasam and Zbib demonstrated how representative volume element (RVE)-based parametric studies can map microstructural choices to macroscopic response in dual-phase steels [107]. In such cases, enhanced strengthening may arise intrinsically from constrained deformation, even when each zone is described by a conventional constitutive law. For gradient materials, discretization into multiple zones can approximate a continuous property profile and reproduce optimal stress-partitioning patterns identified in numerical analyses [116]. In dual-phase or particulate systems, micromechanical homogenization methods provide a complementary strategy in which local stress and strain fields emerge naturally from the spatial distribution of material properties and geometric features.

An essential component of many continuum formulations for heterostructured materials is the incorporation of a back-stress or kinematic hardening term to represent long-range internal stresses arising from mechanical incompatibility. The HDI framework establishes a connection between these internal stresses, the experimentally observable Bauschinger effect, and the additional strain hardening observed in gradient structures and laminates [2,9,63]. Investigations of copper–brass laminates have further examined the quantitative relationship between HDI hardening and imposed strain gradients, indicating that back-stress evolution laws must incorporate physically interpretable saturation mechanisms associated with dislocation structure evolution [86]. For clarity and transferability, constitutive models should explicitly state whether back-stress terms are intended to represent GND pile-ups, interface constraint effects, or a combination, as this distinction influences parameter calibration and applicability across architectures.

Strain-gradient plasticity and related higher-order continuum theories provide a physically motivated framework for linking characteristic heterogeneity length scales to strengthening mechanisms and for regularizing strain localization. Such approaches have been applied to gradient nanograined and heterogeneous microstructures to capture size effects and reproduce dispersed banding behavior [30,32,114]. A persistent modeling challenge is the separation of gradient-induced contributions from kinematic hardening effects. If both mechanisms are introduced without careful distinction, double-counting may occur, as each can represent GND-mediated internal stresses [117]. A practical diagnostic strategy is to compare predicted GND densities or gradient-associated energetic terms with independent estimates derived from EBSD-based lattice curvature analyses, meaning GND-density estimates inferred from spatial gradients of EBSD-measured lattice orientation or dislocation-based models.

Dislocation-density-based continuum formulations offer an alternative representation in which statistically stored dislocations (SSDs) and GNDs evolve as internal state variables. Multi-mechanism constitutive models developed for gradient nanograined materials have demonstrated how dislocation storage, migration, and damage processes can be coupled within a continuum framework [118,119]. Dislocation-mechanism-informed simulations have provided size-dependent insights that can inform the development of such continuum closures when component-scale prediction is required [34,120]. Although dislocation-mechanism-based crystal plasticity models can be reduced to effective continuum descriptions, such reductions must preserve the correct dependence on architectural features and on the microstructural parameters governing mechanical incompatibility to maintain predictive fidelity.

A recurrent modeling decision in continuum descriptions of heterostructures concerns the representation of interfaces and transition zones. Some formulations assume perfectly bonded, sharp interfaces and rely exclusively on kinematic compatibility to generate internal stresses. Others incorporate explicit interfacial elements or graded layers to represent interface-affected zones with finite thickness [121,122,123,124]. For heterogeneous laminates, continuum models that include experimentally measured transition layers have demonstrated improved predictive robustness for both strength and uniform elongation relative to sharp-interface idealizations [125,126,127]. Comparable approaches have been adopted for surface-gradient layers in which residual stress fields and work-hardening gradients coexist [128,129]. These findings underscore the necessity of aligning digital representation with constitutive assumptions to ensure physical consistency.

### 5.3. Model Parameter Identifiability

**Figure 10:**
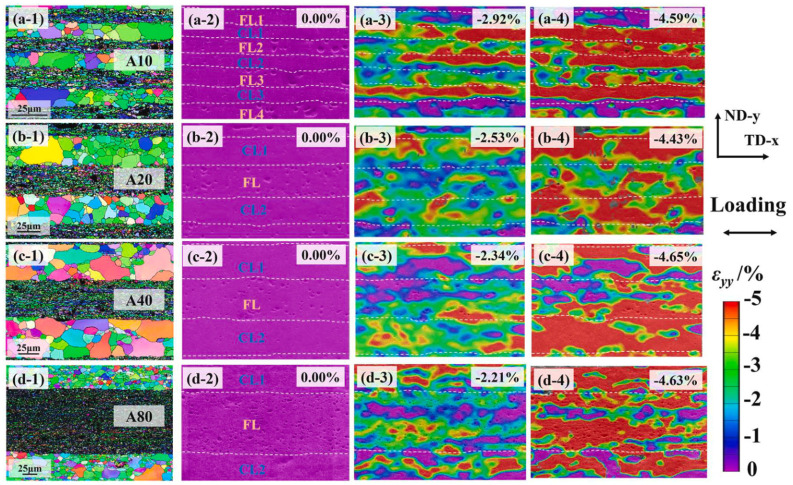
Layer-resolved local strain maps in heterogeneous multilayered aluminum obtained from full-field deformation measurements [130]. The rows correspond to multilayer architectures with different layer thicknesses: (**a-1**–**a-4**) A10, (**b-1**–**b-4**) A20, (**c-1**–**c-4**) A40, and (**d-1**–**d-4**) A80. Subfigures (**a-1**), (**b-1**), (**c-1**), and (**d-1**) present the corresponding microstructure maps, showing the alternating layers and their morphologies. Subfigures (**a-2**), (**b-2**), (**c-2**), and (**d-2**) show the reference configurations with layer labels and interface locations. Subfigures (**a-3**), (**b-3**), (**c-3**), and (**d-3**) display high-resolution digital image correlation (HR-DIC) maps of the axial strain component $\varepsilon_{yy}$ at intermediate applied strains of −2.92%, −2.53%, −2.34%, and −2.21%, respectively. Subfigures (**a-4**), (**b-4**), (**c-4**), and (**d-4**) present the corresponding strain maps at higher applied strains of −4.59%, −4.43%, −4.65%, and −4.63%, respectively. These measurements reveal spatial localization patterns, strain-band continuity across interfaces, and layer-scale strain partitioning, providing valuable calibration and validation targets for both multi-zone continuum models and microstructure-resolved simulations of heterostructured materials.

Parameter identifiability remains a central limitation of many continuum approaches. Additional parameters associated with intrinsic length scales [7,25], back-stress evolution, and inter-zone coupling increase model flexibility but also amplify risks of non-uniqueness. Calibration against a single macroscopic stress–strain curve is generally insufficient. Joint calibration to stress-partitioning data, full-field strain measurements, and Bauschinger response significantly reduces non-identifiability and enhances model transferability [22,28,130]. Full-field strain maps such as Figure 10 are particularly powerful for parameter identification because they constrain both the magnitude and the spatial distribution of localization. Therefore, they reduce non-uniqueness more effectively than bulk response data alone.

Continuum strategies also extend beyond monotonic loading conditions. Rate-dependent and viscoplastic formulations have been applied to model impact and high strain-rate behavior in gradient and layered materials, incorporating thermally activated mechanisms and adiabatic heating effects [131,132,133]. Under cyclic loading, models that couple back-stress evolution, residual stress redistribution, and damage accumulation have been proposed for fatigue assessment in gradient steels and surface-treated alloys. These models demonstrate that predictive accuracy depends critically on the internal stress state established during processing [134,135,136,137]. In such circumstances, calibration requires time-resolved or cycle-resolved field observables rather than reliance solely on stabilized hysteresis loops. Although the calibration burden is substantial, it can be mitigated through hierarchical experimental design and uncertainty-aware parameter estimation.

In general, continuum models are most reliable when their input parameters and intrinsic length scales are directly linked to measured heterogeneity and when validation incorporates at least one internal-field observable. They are least defensible when gradient or kinematic terms function primarily as curve-fitting devices. Rigorous verification is equally essential, as predictions of strain localization may exhibit mesh dependence in the absence of appropriate regularization [30,35]. Accordingly, best practices include explicit documentation of boundary conditions, mesh resolution relative to heterogeneity length scales, and results of sensitivity analyses.

In summary, continuum multi-zone models offer computational efficiency and architectural flexibility, but their predictive reliability is often undermined by parameter non-identifiability and conceptual double-counting. Many studies calibrate kinematic hardening or strain-gradient parameters exclusively against macroscopic stress–strain curves, producing mathematically correct but mechanistically ambiguous representations in which back-stress evolution cannot be uniquely attributed to GND pile-ups, interface constraints, or numerical regularization. The field lacks systematic intercomparisons of how different continuum formulations perform when validated against the same internal-field datasets, such as diffraction-derived stress partitioning or EBSD-based GND maps. Without such benchmarks, the apparent success of a given model often reflects calibration flexibility rather than physical fidelity, limiting transferability across architectures or loading conditions.

## 6. Crystal Plasticity Modeling of Heterostructures

Crystal plasticity frameworks resolve deformation at the scale of individual grains and their associated slip or twinning systems, which make them particularly well suited for the analysis of heterostructures where crystallography and texture strongly influence stress partitioning and strain localization. Crystal plasticity finite element method (CPFEM) investigations of nanodomained heterogeneous architectures and bicrystalline thin films have demonstrated that, even at identical phase fractions, zone-dependent anisotropy can reorganize strain localization patterns and alter the predicted onset of necking [138,139]. These findings underscore an important consideration for model development: apparent mechanical heterogeneity is governed not solely by strength contrast, but also by the distribution of crystallographic orientations and their spatial correlations across hetero-zones [140,141].

### 6.1. Crystal Plasticity Formulations

The kinematic basis of many crystal plasticity formulations is the multiplicative decomposition of the deformation gradient,

(6) $$F=F^{e}F^{p},$$

with $F^{p}$ arising from plastic slip and $F^{e}$ representing elastic stretch and rotation in an intermediate configuration [142]. Plastic flow is described through the plastic velocity gradient,

(7) $$L^{p}={\dot{F}}^{p}F^{p-1}=\sum_{\alpha }{\dot{\gamma }}^{\alpha }(s^{\alpha }⊗m^{\alpha }),$$

where $s^{\alpha }$ and $m^{\alpha }$ denote the slip direction and slip-plane normal, respectively, for slip system α. Slip-system-based formulations establish a direct connection between crystallographic deformation mechanisms, texture evolution, and rate-dependent hardening in polycrystalline aggregates [143]. Rate-dependent power-law kinetics are widely employed, commonly expressed as [144]:

(8) $${\dot{\gamma }}^{\alpha }={\dot{\gamma }}_{0}{|\frac{\tau^{\alpha }}{g^{\alpha }}|}^{1/m}sign(\tau^{\alpha }),$$

where $\tau^{\alpha }$ is the resolved shear stress, $g^{\alpha }$ is the slip resistance, ${\dot{\gamma }}_{0}$ is a reference shear rate, and m is the rate-sensitivity exponent. The magnitude of rate sensitivity has been shown to exert a strong influence on the onset and evolution of localized deformation in crystalline solids, highlighting the importance of carefully calibrating m, when evaluating heterostructures across varying strain rates [145]. When coupled with thermally activated hardening and recovery mechanisms, such formulations can capture strain-rate sensitivity and temperature-dependent behavior in heterogeneous alloys [145]. For the essential equations of CPFEM, readers are referred to this reference [146].

### 6.2. Crystal Plasticity Models Tailored for Heterostructures

**Figure 11:**
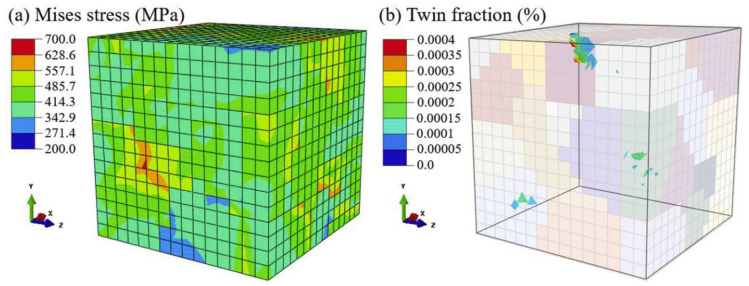
CPFEM contour plots of Mises stress (**a**) and twin volume fraction (**b**) in gradient TWIP steel at 0.25% engineering strain [33]. This figure shows how local stress concentration and twinning activity distribute nonuniformly across the gradient, thereby shaping the overall hardening response.

In heterostructured materials, crystal plasticity formulations must incorporate zone-dependent constitutive descriptions rather than relying on a single global parameter set. Depth-dependent variations in deformation mechanisms, slip resistance, and hardening behavior are often essential to reproduce experimentally observed responses. For example, simulations of gradient TWIP steels that incorporated spatially varying twinning activity and grain-size-dependent slip resistance successfully captured how microstructural gradients modify the sequence of operative mechanisms and sustain hardening beyond rule-of-mixtures predictions [33]. Strain-gradient plasticity finite element analyses of Cu-Fe layered systems have further demonstrated how interfacial constraint influences the evolution of stress and strain partitioning as functions of layer thickness and strength contrast [147]. Related full-field simulations indicate that the temporal evolution of stress partitioning, rather than merely its initial magnitude, determines whether hard layers stabilize plastic flow or instead accelerate interface shear and premature localization [84,148]. These findings feature the importance of capturing spatially resolved mechanism transitions and evolving constraint effects in predictive models of heterostructures. The advantage of tailored CPFEM formulations is that they reveal where mechanisms activate and how constraint evolves spatially, not just when yielding begins. Figure 11 illustrates this point through local fields of stress and twin volume fraction.

Physically based hardening formulations link slip resistance, $g^{\alpha }$, to evolving dislocation densities. The classical Taylor relation between flow strength and dislocation density provides the foundation for Taylor-type hardening laws of the form [149]

(9) $$g^{\alpha }=\tau_{0}^{\alpha }+a\mu b\sqrt{\sum_{\beta }h_{\alpha \beta }\rho^{\beta }},$$

with initial resistance $\tau_{0}^{\alpha }$, constant a, shear modulus μ, Burgers vector b, and latent hardening matrix $h^{\alpha \beta }$. Evolution equations for dislocation density, $\rho^{\beta }$, include storage and dynamic recovery terms [34,120]. Size-dependent crystal plasticity models incorporating explicit dislocation mechanisms have demonstrated that grain-size-dependent mean free paths and recovery kinetics are required to reproduce both yield strength trends and post-yield hardening across gradient nanograined copper architectures [34]. Nonlocal crystal plasticity formulations have further connected GND density to kinematic hardening in gradient-grained materials, reducing sensitivity to mesh discretization when sharp gradients are present [120].

Nonlocal hardening variants that incorporate GND storage and transport can improve representation of gradient-induced kinematic effects; however, their reliability depends on validation against independent estimates of GND density and experimentally observed unloading–reloading behavior [117,150]. A principal advantage of crystal plasticity in heterostructure modeling is the quantitative prediction of GND density fields and associated HDI stress proxies. Lattice-curvature-consistent GND measures derived from nonlocal or strain-gradient crystal plasticity can be compared directly with EBSD curvature analyses and microdiffraction-based lattice rotation measurements [120,151]. In gradient nanotwinned copper, inclusion of multiple dislocation populations and gradient terms clarified the persistence of strengthening even after conventional dislocation storage mechanisms saturate, while enabling separation of genuine size effects from numerical smoothing artifacts near sharp gradients [120,152]. Comparative studies of back-stress quantification further indicate that predicted HDI stresses must be consistent with experimentally observed tension–compression asymmetry and unloading signatures [150,153].

Interfaces and transition layers exert a strong influence on the transferability of CPFEM simulations. In medium-Mn steels, combined experimental and modeling investigations have shown that austenite-ferrite interfaces govern yielding through localized constraint and load-transfer mechanisms, motivating interface descriptions that differentiate phase-specific slip and transformation contributions [52]. CPFEM implementations range from idealized perfect bonding, where compatibility alone generates internal stress, to formulations incorporating explicit interface elements or slip-transfer resistance laws that account for interfacial penetrability and barrier strength [154]. Comparisons between micromorphic crystal plasticity and discrete dislocation dynamics (DDD) for multilayer pile-up hardening have demonstrated that appropriately calibrated interface constraint parameters can reproduce mesoscale pile-up signatures without explicitly resolving individual dislocations [37].

### 6.3. Solver Selection and Model Reliability

Solver selection imposes practical tradeoffs that can influence predicted strain gradients and localization patterns. Spectral crystal plasticity approaches based on the crystal plasticity fast Fourier transform (CPFFT) approach offer substantial computational efficiency for periodic RVEs and are therefore well suited to ensemble simulations addressing microstructural uncertainty, including bimodal polycrystals and heterogeneous lamellar systems [35,155]. In contrast, CPFEM provides greater flexibility for modeling free surfaces, complex specimen geometries, and experimentally informed boundary conditions, such as conditions frequently encountered in gradient surface layers and laminate coupon tests. In both classes of solvers, sharp property transitions demand careful numerical treatment. Excessive interpolation across hetero-zone boundaries may artificially smooth stress gradients, leading to underprediction of peak internal stresses and, consequently, HDI hardening.

Crystal plasticity modeling becomes substantially more informative when validated against spatially resolved field observables rather than solely against macroscopic stress–strain curves. Three-dimensional X-ray microdiffraction has been employed to map submicron-scale heterogeneity. It can provide direct quantitative targets for predicted lattice-strain distributions and their evolution with applied strain [28]. In situ interfacial observations combined with microstructure-sensitive analysis have further enabled validation of interface-controlled dislocation activity within crystal plasticity frameworks [29]. Additionally, diffraction-based measurements of stress partitioning in multilayered steels and localized strain and fracture observations in heterogeneous multilayered aluminum motivate validation strategies that incorporate band topology and correlation lengths in addition to global mechanical response [22,130]. Studies reporting agreement only at the level of overall stress–strain curves risk parameter non-identifiability, particularly when zone-specific hardening parameters and interface constraint descriptions are adjusted simultaneously.

Overall, crystal plasticity approaches are most effective when the governing deformation mechanisms are crystallographic in nature and when texture evolution and slip or twinning activity dictate strain localization and the emergence of damage precursors. Their applicability is more limited in regimes dominated by amorphous plasticity, grain-boundary-mediated mechanisms at the nanoscale, or progressive interface degradation requiring explicit decohesion formulations. In practical implementation, several issues are decisive: which zone-dependent slip and twinning systems must be activated; whether explicit evolution of GNDs is required to HDI stresses; how slip transfer and barrier effects are represented at interfaces; and which internal-field observables are employed to constrain calibration. Recent efforts integrating crystal plasticity with explicit damage models and phase-field fracture formulations are beginning to address these limitations for gradient composites and layered heterostructures [102].

A consistent conclusion emerging from recent studies is that physically grounded parameterizations enhance predictive extrapolation. Dislocation-informed crystal plasticity simulations of grain-size-dependent behavior in dual-phase steels and gradient architectures indicate that apparent strengthening may arise from cooperative plasticity mechanisms spanning multiple grains and hetero-zones, rather than solely from local Hall–Petch-type scaling [156,157]. Consequently, the reliability of crystal plasticity models for heterostructured materials increasingly depends on demonstrating that predicted internal fields, including stress partitioning patterns and GND distributions, remain quantitatively reliable when geometry, loading mode, or characteristic length scale is varied.

In summary, crystal plasticity models provide the crystallographic resolution necessary to capture texture-sensitive stress partitioning and slip/twinning-mediated localization. Yet a recurring limitation is the oversimplification of interface physics: most implementations assume perfect bonding and rely on compatibility alone to generate internal stresses, neglecting slip-transfer resistance, interface decohesion, or the finite thickness of boundary-affected zones. Where interface elements or transmission laws are incorporated, their parameters are rarely constrained by independent measurements but are instead treated as fitting parameters. Furthermore, validation against full-field strain maps or lattice rotation data remains the exception rather than the norm. Many studies claim predictive success based solely on macroscopic curve agreement, which is insufficient to discriminate among competing slip-system activation or hardening formulations. Advancing the field requires routine reporting of spatially resolved validation metrics and systematic sensitivity analyses to interface parameter uncertainty.

## 7. Dislocation-Based Mesoscale Approaches

**Figure 12:**
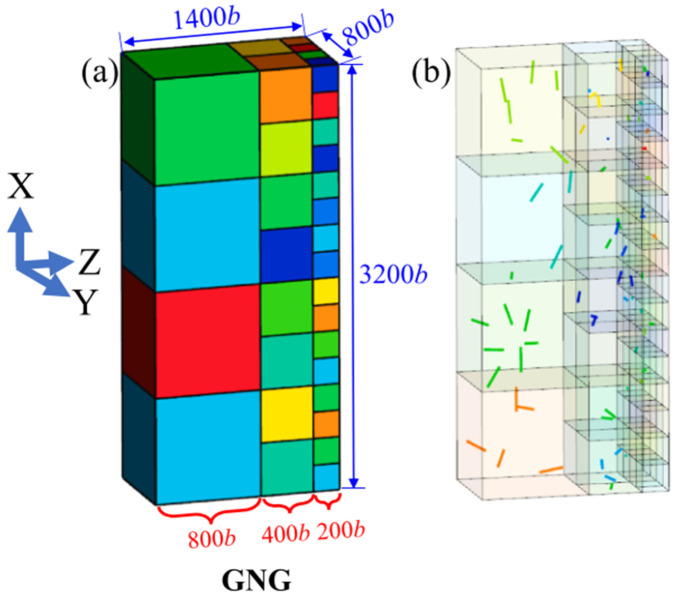
Computational geometries used in discrete dislocation simulations of gradient nanograined materials and corresponding reference cases. (**a**) Gradient nano-grained (GNG) model with (**b**) initial FR source distribution in the whole volume [36].

Dislocation-based mesoscale approaches capture incompatibility-driven strengthening and strain hardening by explicitly or statistically resolving the dislocation structures that develop in the vicinity of hetero-zone boundaries. Figure 12 introduces the mesoscale dislocation dynamics simulation setting by showing the computational geometries used to study gradient nanograined materials and their homogeneous counterparts. These methods are particularly valuable when the objective is mechanistic understanding, such as elucidating the formation of dislocation pile-ups at interfaces, exploring the dependence of transmission and reflection processes on interfacial character, or the role of dislocation transport in governing the thickness and evolution of boundary-affected regions [36,158,159].

### 7.1. Discrete Dislocation Dynamics (DDD) Simulation

A fundamental aspect of DDD simulations is the accurate computation of the stress field generated by every dislocation segment, as this internal stress, superimposed with any applied load, dictates the Peach-Koehler force on all other dislocations. For a general three-dimensional network, dislocations are discretized into a series of straight or curved segments [160]. The stress field $\sigma^{\mathrm{seg}}(x)$ at a material point ***x*** due to a single straight dislocation segment from point ***p*** to ***q*** with Burgers vector ***b*** can be derived from the displacement field of a dislocation loop [161]. For a segment, the stress is a function of the position vector ***R*** = ***x*** − ***ξ*** along the segment and its line direction ***t***. The contribution involves terms proportional to 1/R, leading to the characteristic long-range nature of dislocation interactions. The total internal stress field $\sigma^{\mathrm{int}}(x)$ at any point is then the sum of the contributions from all *N* dislocation segments in the simulation cell [162,163]:

(10) $$\sigma^{\mathrm{int}}(x)=\sum_{i=1}^{N}\sigma_{i}^{\mathrm{seg}}(x).$$

The complete stress field $\sigma^{\mathrm{total}}(x)$ experienced by a dislocation segment is the superposition of this internal field and the externally applied stress $\sigma^{\mathrm{app}}$:

(11) $$\sigma^{\mathrm{total}}(x)=\sigma^{\mathrm{app}}(x)+\sigma^{\mathrm{int}}(x).$$

This total stress is then used to compute the Peach-Koehler force on each dislocation segment. For a straight segment with Burgers vector b and line sense vector ξ, the force per unit length is [164]

(12) $$F=(\sigma^{\mathrm{total}}\cdot b)\times \xi .$$

**Figure 13:**
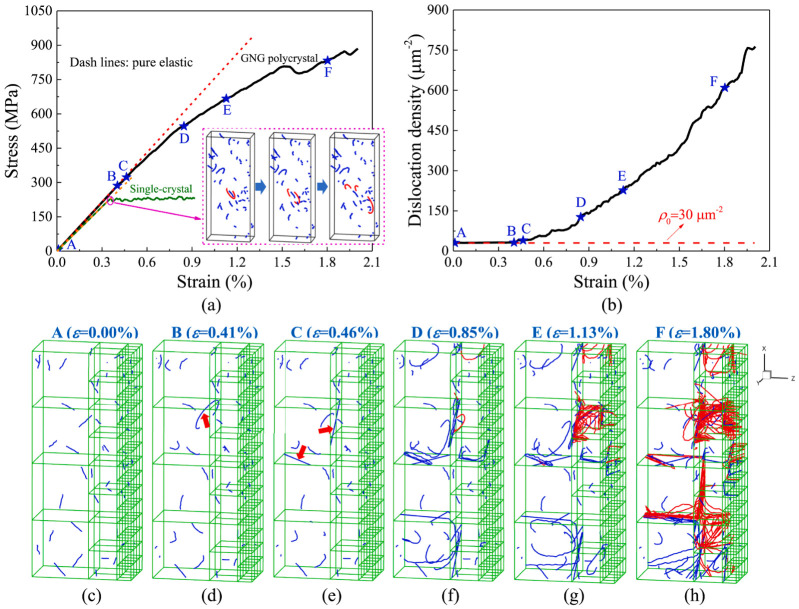
DDD-predicted stress–strain response together with evolving dislocation structures in a gradient nanograined architecture [36]. (**a**) Stress–strain curve, (**b**) dislocation density-strain curve and (**c**–**h**) corresponding dislocation structure evolution of GNG samples. The figure links macroscopic hardening to explicit mesoscale events such as source activation, pile-up accumulation, and interaction with hetero-zone boundaries. (The red arrows in (**d**) and (**e**) indicate the first dislocation activation event and the dislocation pile-ups at grain boundaries (GBs), respectively. Dislocations emitted from sources within the grain interior are shown in blue, whereas those emitted from grain boundaries are represented by red lines).

When combined with mobility laws that encode drag and temperature dependence, DDD simulations can resolve interface pile-ups, source activation, and the statistics of transmission events [165]. Figure 13 demonstrates the key strength of DDD: it simultaneously predicts the macroscopic response and the evolving defect structure that produces it.

Lu et al. conducted DDD simulations in gradient and layered metals and demonstrated that pre-existing residual stresses and inherited dislocation structures can alter the apparent yielding sequence [166]. Long et al. reported that such internal stresses can contribute to tension-compression asymmetry even before large additional GND storage develops in gradient nanograined copper [63]. Synergetic deformation induced strengthening in gradient nano-grained metals has also been quantified by three-dimensional DDD simulations. In these simulations, dislocations migrate toward boundaries and form pile-ups whose back stress scales with gradient length and layer thickness [158]. Further analyses indicate that thin layers may exhibit pile-up-controlled strengthening when dislocation sources are limited, while thicker layers tend to transition toward storage-dominated hardening during continued plastic straining [36].

Despite their mechanistic value, representativeness and dimensionality remain significant constraints in DDD-based approaches. Two-dimensional simulations tend to exaggerate pile-up coherence and suppress critical dislocation reaction mechanisms, such as cross-slip and junction formation, thereby potentially overpredicting strengthening relative to fully three-dimensional simulations for equivalent interface barrier strengths [36,158]. As a matter of best practice, scaling relations derived from DDD simulations should be validated against at least one three-dimensional dataset or against independent experimental field observables, such as lattice curvature measurements or back-stress signatures, before being incorporated into continuum-scale constitutive models.

### 7.2. Continuum Dislocation Dynamics (CDD) Modeling

CDD models replace discrete dislocation lines with continuous dislocation density fields, thereby providing an intermediate description that bridges DDD simulations and phenomenological continuum constitutive formulations. While early CDD models often treated dislocation densities as scalar internal variables, modern CDD provides a more rigorous, field-theoretic framework by introducing higher-dimensional dislocation density tensors that preserve information about dislocation line orientation and curvature [167,168]. This allows CDD to capture critical mesoscale phenomena such as the formation of dislocation patterns, the evolution of curvature, and the transport of dislocations across hetero-zone boundaries, which are essential for modeling gradient and laminate structures.

A central quantity in CDD is the dislocation density tensor field ρ(x,t), which represents the local distribution of dislocation lines. For a population of dislocations, this can be decomposed into orientation-specific densities. The evolution of these densities is governed by a set of conservation laws in a generalized configuration space that includes both spatial coordinates and an additional variable for line orientation. A simplified, yet powerful, formulation for curved dislocations introduces the dislocation density ρ(x,t) and the curvature density q(x,t) as key field variables [169]. The evolution equations for a system of curved dislocations gliding on a single slip system can be written as:

(13) $$\partial_{t}\rho +\nabla \cdot \left(\rho v\right)=\rho vk,\;\;\;\;\;\;\partial_{t}q+\nabla \cdot (qv)=\rho vk^{2},$$

where v=vs is the dislocation velocity vector (with s the line direction), and k is the local curvature. The source term, ρvk, represents the change in dislocation density due to the expansion or contraction of curved loops. This term is a hallmark of CDD models, capturing the fact that an expanding circular loop increases its total line length. To close this system, constitutive relations are required for the velocity v as a function of the local effective stress $\tau_{\mathrm{eff}}$, which includes the resolved shear stress, a back stress $\tau_{b}$ from dislocation interactions, and a friction stress. The back stress itself can be derived from the gradient of the dislocation density tensor, linking it directly to the incompatibility of plastic strain and the GND content [34,170]. For multiple slip systems, the CDD framework becomes a system of coupled transport-reaction equations, with terms accounting for dislocation reactions (e.g., junction formation, annihilation) that depend on the densities and orientations of interacting slip systems [171].

In heterostructured materials, CDD and related dislocation-density-based approaches yield two categories of outputs that are particularly relevant for higher-scale modeling. First, they predict spatial distributions of GND density in the vicinity of interfaces and across gradient regions. These predictions can be compared directly with EBSD curvature-based proxies and can inform calibration of kinematic hardening terms in conventional continuum models [170,172]. Second, the inclusion of transport terms permits explicit representation of dislocation flux toward and across hetero-zone boundaries, which is essential when boundary-affected regions evolve with increasing strain or under cyclic loading conditions [37,172].

A common multiscale strategy involves using DDD and CDD results to inform interface and hardening laws in CPFEM and continuum constitutive frameworks. For example, distributions of transmission stresses extracted from DDD simulations may be translated into slip-transfer criteria or interface yield conditions, while CDD-derived GND energy contributions can provide physically motivated regularization of strain localization without reliance on purely numerical smoothing [37,120]. In general, dislocation-based methods are powerful for mechanistic discovery and derivation of scaling relationships. Their principal limitation lies in the computational expense and in the uncertainty associated with transferring statistics obtained from idealized simulations to complex, experimentally characterized microstructures.

In summary, discrete and continuum dislocation dynamics provide mechanistic insights into pile-up formation, transmission statistics, and gradient-length-scale effects that are inaccessible to higher-scale models. However, the transferability of DDD results to engineering microstructures is constrained by idealized geometries (e.g., single-slip, planar interfaces, periodic arrays) and by the extreme computational cost that limits three-dimensional simulations to small volumes and short times. The prevalent use of two-dimensional DDD remains problematic, as it artificially enforces dislocation parallelism and suppresses cross-slip and junction formation, potentially overpredicting back stresses. While CDD offers a more scalable field-theoretic alternative, its adoption in the heterostructure community has been limited, and systematic comparisons between CDD predictions and experimental GND density maps are nearly absent. A critical priority is the development of openly shared DDD and CDD benchmark problems, calibrated against well-characterized laminate or gradient specimens, to establish which mesoscale phenomena must be explicitly resolved versus those that can be safely homogenized.

## 8. Atomistic and Interface-Focused Modeling

**Figure 14:**
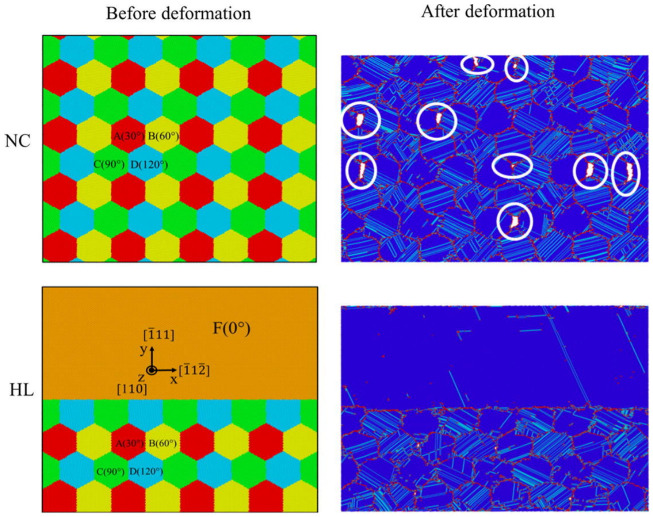
Atomistic modeling of the deformation in homogeneous nanocrystalline Ni and heterogeneous nanocrystalline Ni lamellae [38]. The **left** figure defines the nanoscale structural models employed to study defect activity, interface response, and failure mechanisms. The **right** figure illustrates the predicted crack-resistance advantage of heterogeneous nanocrystalline Ni lamellae (**bottom**) relative to homogeneous reference (**top**) structures. This figure shows how nanoscale interface interactions modify stress concentration, crack advance, and local defect activity near the crack tip. For each grain, the atom color represents its crystallographic orientation. Atoms are also classified according to their common neighbor analysis (CNA) values: FCC atoms are shown in dark blue, HCP atoms in light blue, and atoms with unidentified local structures in red. Voids and cracks are highlighted by circles.

Atomistic simulations, mostly implemented through molecular dynamics (MD) simulations, provide direct insight into nanoscale mechanisms that govern heterostructure behavior, as many critical deformation events occur within nanometer-scale boundary regions. Simulations of heterogeneous nanocrystalline nickel lamellae have resolved dislocation emission, absorption, and grain-boundary-mediated processes during loading, demonstrating how such interface-localized events redistribute strain between adjacent lamellae and establish the initial HDI strengthening response [38]. Atomistic tensile simulations of gradient nanograined iron have further shown that differential grain-boundary activity across the gradient can generate internal strain gradients and early localization precursors, even under nominally uniform external loading conditions [173]. Figure 14 introduces the atomistic configurations used to isolate interface effects in heterogeneous nanocrystalline Ni lamellae. One of the main values of atomistic modeling is that it can reveal how interface-mediated interactions alter local failure resistance before such effects are accessible to continuum models [38].

In MD, atomic trajectories are obtained by integrating Newton’s equations of motion for atoms interacting via an interatomic potential. For metallic systems, embedded-atom method (EAM) potentials [174] are frequently employed, with total energy expressed as

(14) $$U=\sum_{i}F_{i}(\rho_{h,i})+\frac{1}{2}\sum_{i\neq j}\phi_{ij}(r_{ij}),$$

leading to the equations of motion

(15) $$m_{i}\frac{d^{2}r_{i}}{dt^{2}}=-\nabla_{r_{i}}U.$$

In Equation (14), U represents the total potential energy of the entire atomic system. The term $F_{i}$ denotes the embedding energy function, which quantifies the energy required to place atom *i* into the surrounding electron cloud, while $\rho_{h,i}$ stands for the host electron density at the site of atom *i* contributed by all neighboring atoms. The pairwise interaction between any two atoms *i* and *j* is governed by the potential function $\phi_{ij}$, which depends on $r_{ij}$, the physical distance separating those two atoms. The indices *i* and *j* simply serve as labels to identify individual atoms within the simulation system.

In Equation (15), which expresses Newton’s second law of motion for atomic dynamics, $m_{i}$ indicates the mass of atom *i*, and $r_{i}$ represents its position vector in three-dimensional space. The expression $\frac{d^{2}r_{i}}{dt^{2}}$ describes the acceleration of atom *i*, calculated as the second derivative of its position with respect to time *t*. On the right side of the equation, $\nabla_{r_{i}}$ is the gradient operator taken with respect to the position of atom *i*, and when applied to the total potential energy U, its negative value ($-\nabla_{r_{i}}U$) yields the net interatomic force acting on that atom, thereby determining how its trajectory evolves over time.

This formalism enables direct observation of dislocation nucleation and interface reactions that are challenging to resolve experimentally at small strains [38,174,175,176]. Representative atomistic outputs include interface-specific transmission stresses, probabilities of dislocation absorption versus reflection, and temperature-dependent trends in interface-mediated processes. Such mechanistic insights rationalize sustained hardening through repeated dislocation-interface interactions [29,177] and provide physically grounded parameters for DDD simulations and crystal plasticity interface formulations [37].

The most transferable outcomes of atomistic simulations are interfacial properties that can parameterize higher-scale models. However, transferability is inherently constrained by sensitivity to interface character, including crystallographic orientation relationships, chemical segregation, and defect populations introduced during processing [177,178]. Atomistic simulations also provide a basis for calibrating interface laws employed in damage and fracture models. Traction-separation relationships extracted from molecular dynamics can be fitted to cohesive-zone parameters, while temperature-dependent transmission stresses may be translated into slip-transfer barriers for mesoscale and continuum models [37,179]. Because practical heterostructures comprise distributions of interfaces rather than a single idealized boundary, individual atomistic simulations are seldom representative. Robust parameter export therefore requires sampling across relevant interface character space and propagating the resulting statistical distributions into higher-scale predictions [7,25].

Beyond parameterization, atomistic studies can guide architectural strategies aimed at mitigating incompatibility. For example, gradient nanolayer configurations and substrate-constrained nanocrystalline films have demonstrated that interfacial spacing and external constraint can promote plastic delocalization and delay the onset of catastrophic localization [180,181]. Translating such insights to the microscale necessitates systematic mapping between atomistic length scales and the statistical distribution of interfaces in engineering microstructures.

A fundamental limitation of atomistic modeling arises from accessible time scales, which necessitate strain rates far exceeding those used in most experiments. This discrepancy is particularly consequential for thermally activated mechanisms, including dislocation climb, diffusion-mediated interface weakening, and segregation-driven modifications of barrier strength [182,183]. Basically, atomistic approaches are most powerful for bounding interface constitutive laws and elucidating governing mechanisms, and least suited for direct quantitative prediction in the absence of a validated scale-bridging framework.

In summary, atomistic simulations have uniquely resolved dislocation–interface interactions, transmission mechanisms, and incipient plasticity at hetero-zone boundaries. Their primary value lies in providing physically grounded bounding estimates for higher-scale interface laws, such as slip-transfer resistances or cohesive-zone parameters. However, the strain-rate gap (typically $10^{7}$–$10^{9}$ s^−1^ in MD vs. $10^{-3}$–$10^{-3}$ s^−1^ in experiments) and the limited length scales (tens of nanometers) raise fundamental questions about the direct quantitative transferability of atomistic predictions to microscale deformation, particularly for thermally activated or diffusion-mediated processes. Moreover, most atomistic studies examine idealized, defect-free interfaces with perfect crystallographic registry, whereas real heterostructures contain processing-induced defects, segregants, and variable interface character. The community lacks systematic protocols for sampling interface character distributions and propagating the resulting statistical variability into mesoscale and continuum models. Until such scale-bridging frameworks are validated, atomistic simulations should be viewed as mechanistic hypothesis generators rather than as direct quantitative predictors of engineering behavior.

## 9. Damage and Fracture Models for Heterostructures

Damage and fracture modeling is crucial in the analysis of heterostructured materials, as the same incompatibility-driven internal fields that enhance work hardening may also concentrate stress and strain at interfaces, thereby accelerating failure. Experimentally observed failure mechanisms include ductile void nucleation and growth within mechanically softer regions [21], interfacial decohesion in dissimilar laminates [91], and shear-band-induced cracking when strain delocalization collapses into dominant localization [184]. Conversely, heterogeneity may promote crack deflection and spatial dispersion of damage, contributing to enhanced toughness in certain heterogeneous lamellar and gradient architectures [185,186]. A central design consideration is whether heterogeneity acts to toughen or embrittle the material system. In lamellar and gradient configurations, crack deflection and distributed plasticity can elevate fracture resistance, whereas abrupt property transitions may intensify shear localization and precipitate interfacial cracking [187,188]. Numerical studies suggest that the ultimate outcome depends on the strain-dependent evolution of stress partitioning and the competition between interfacial decohesion processes and bulk ductile damage mechanisms [185,189].

Among the studies of damage and fracture of heterostructured materials, cohesive-zone models (CZM) and phase-field fracture (PFF) models are widely used, and they are built upon distinct philosophical foundations. The most fundamental difference lies in how they represent a crack. CZM treats a crack as a discrete surface of discontinuity. Among the studies of damage and fracture of heterostructured materials, cohesive-zone models (CZM) and phase-field fracture (PFF) models are widely used, and they are built upon distinct philosophical foundations. The most fundamental difference lies in how they represent a crack. CZM treats a crack as a discrete surface of discontinuity. Fracture is confined to predefined interfaces or potential crack paths, where cohesive elements are inserted [190,191,192]. This makes CZM conceptually intuitive and efficient when crack paths are known as a priori, such as delamination along a weak interface or debonding of a reinforcing particle.

In CZM, the behavior of the crack is governed by a traction-separation law (TSL) that relates the traction vector T across the interface to the displacement jump Δ. For a simple exponential form, the normal traction is:

(16) $$T_{n}=\frac{\phi_{n}}{\delta_{n}}\left(\frac{\Delta_{n}}{\delta_{n}}\right)\;\exp\left(-\frac{\Delta_{n}}{\delta_{n}}\right)\;\exp\left(-\frac{\Delta_{t}^{2}}{\delta_{t}^{2}}\right),$$

where $\phi_{n}$ is the normal work of separation, $\delta_{n}$ and $\delta_{t}$ are characteristic separation lengths in the normal and tangential directions, and $\Delta_{n}$ and $\Delta_{t}$ denote the corresponding displacement jumps [192]. This is a local, path-dependent law. Numerically, CZM requires the insertion of cohesive elements along element boundaries in a finite element mesh. Model parameters may be calibrated from dedicated interface experiments or informed by atomistic simulations [179].

**Figure 15:**
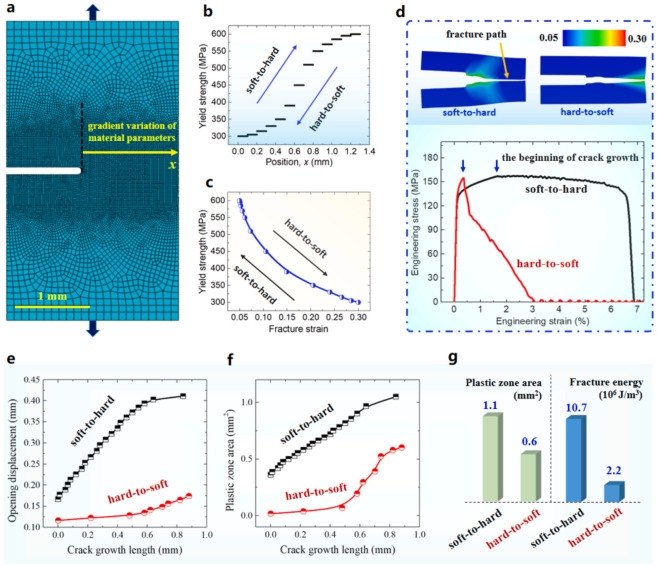
Finite-element fracture simulation of a gradient structure with evolving local strength/ductility and crack-tip plasticity [199]. (**a**) Finite element model. (**b**,**c**) Variations in yield strength and fracture strain along the crack line. (**d**) The contour map of equivalent plastic strain and the tensile stress–strain curves. (**e**) The crack opening displacement at the free surface during crack growth. (**f**) The total area of plastic zone during crack growth. (**g**) The total area of plastic zone and fracture energy. This figure illustrates how a property gradient alters crack-driving force, plastic-zone development, and the predicted fracture response.

In contrast, PFF models regularize the sharp crack by smearing it over a small but finite length scale $l_{0}$, introducing a continuous phase field variable d(x,t)∈[0,1] that represents the state of damage [193,194]. A crack is not a discrete entity but a diffuse region where d approaches 1. This diffusive representation is a strength when crack paths are complex, branching, or unknown in advance, as the phase field can evolve naturally without any need for remeshing or predefined paths. The PFF models are rooted in variational principles of fracture mechanics. The total energy functional, the total potential energy, may be expressed as

(17) $$\Pi (u,d)=\int_{\Omega }\left[\left(1-d{)}^{2}\psi^{+}(\varepsilon )+\psi^{-}(\varepsilon \right)\right]\;d\Omega +\int_{\Omega }G_{c}\left(\frac{d^{2}}{2l_{0}}+\frac{l_{0}}{2}|\nabla d{|}^{2}\right)d\Omega ,$$

where $\psi^{+}$ and $\psi^{-}$ denote the tensile and compressive strain energy densities, $G_{c}$ is the critical energy release rate, and $l_{0}$ is a regularization length scale [193]. This is a non-local model due to the gradient term $|\nabla d{|}^{2}$. The evolution of the phase field is governed by a partial differential equation derived from the variational principle, which is solved simultaneously with the mechanical equilibrium equations. This coupled system can be implemented in standard finite element codes without the need for embedded discontinuities, but it requires a mesh fine enough to resolve the length scale $l_{0}$ [194]. Crystal-plasticity phase-field simulations applied to particle-reinforced gradient composites have demonstrated how gradients redistribute crack-driving forces and promote crack deflection, thereby influencing toughness [102]. Figure 15 complements the conceptual schematic by showing how a coupled fracture model redistributes crack-driving force once local strength and ductility vary along the gradient.

For modeling heterostructured materials, each approach offers distinct advantages. CZM excels at simulating failure along well-defined interfaces, such as phase boundaries in laminates, grain boundaries in gradient structures, or the interface between a hard coating and a soft substrate. The parameters of the TSL (cohesive strength, fracture energy) have clear physical interpretations and can be directly informed by atomistic simulations or micromechanical experiments [176,195]. PFF is uniquely powerful for predicting complex crack patterns in microstructures with unknown failure paths. In a heterostructured material with a random distribution of hard and soft phases, the phase field can naturally capture crack initiation at stress concentrators, crack deflection around obstacles, and crack branching without any a priori assumptions [102,196]. This makes it ideal for studying how heterogeneity descriptors influence crack paths.

Across both methodologies, critical considerations include parameter identifiability, appropriate treatment of mode mixity, and validation of predicted crack paths against spatially resolved experimental observations. Parameter identifiability frequently constitutes a limiting factor. Interface toughness and cohesive strengths are seldom uniquely determined from macroscopic failure strain alone. Integrating fracture tests with full-field strain mapping and post-mortem crack initiation analyses can substantially constrain model calibration. Therefore, they can reduce compensatory errors between plasticity regularization parameters and fracture properties [22,130].

Regularization embedded within plasticity formulation interacts strongly with fracture predictions. In gradient-structured metals, higher-order or strain-gradient plasticity modifies the near-tip plastic zone, thereby influencing whether cracks undergo blunting or transition into shear-band-dominated localization [197,198,199]. Accurate representation of the strain-dependent evolution of stress partitioning is essential, as preferred damage nucleation sites may migrate from softer zones toward interfaces with increasing constraint [83,103]. Under cyclic and dynamic loading, additional coupling arises among heterogeneity, residual stress evolution, and damage processes. Gradient surface layers have been shown to suppress surface crack initiation and retard fatigue crack growth; such effects have been modeled by combining cyclic plasticity formulations with damage or cohesive-zone descriptions [200,201]. At elevated strain rates, adiabatic heating and rate sensitivity can intensify shear localization and promote interfacial failure, motivating the use of coupled thermo-viscoplastic and fracture-regularized frameworks [92].

Overall, damage and fracture models can be assessed according to their ability to predict (i) the spatial location of damage initiation, (ii) the response of crack paths to evolving stress partitioning, and (iii) the conditions under which heterogeneity enhances or diminishes toughness. Essential inputs include interface toughness or cohesive parameters, zone-dependent ductile damage parameters, and at least one spatially resolved field observable for validation. Model credibility is compromised when fracture parameters are tuned solely to reproduce a global failure strain without capturing the underlying localization topology that governs crack initiation and propagation.

## 10. Model Calibration, Validation, and Data Sharing

Calibration and validation of heterostructure models should proceed sequentially, with verification preceding validation. Verification encompasses code correctness, mesh and time-step convergence, and targeted assessments of sensitivity to localization and higher-order boundary conditions. This stage is particularly critical for strain-gradient plasticity and phase-field fracture formulations, as numerical discretization interacts directly with intrinsic regularization length scales [30,198]. Verification results should be documented not only for global responses but also for field quantities, including peak strain gradients, strain-band spacing, and near-interface GND distributions. Parameter identification strategies must be aligned with the model outputs of interest. Calibration to a single macroscopic stress–strain curve is rarely sufficient for multi-zone [7,25], gradient, or damage-coupled models. Joint calibration to macroscopic curves, Bauschinger response, stress-partitioning data, and full-field strain maps substantially reduce parameter non-identifiability.

For clarity, the velocity gradient defines the local deformation rate and is commonly decomposed into elastic and plastic parts in finite-strain plasticity. In crystal plasticity, the plastic velocity gradient is built directly from the slip rates on active systems and therefore provides a mechanistic bridge between measured macroscopic stress–strain curves and the underlying crystallographic kinetics. In practice, macroscopic stress–strain data are useful for an initial calibration of baseline flow parameters and rate sensitivity, but heterostructure-specific parameters, such as intrinsic length scales and inter-zone coupling terms, require additional internal-field validation targets as emphasized below.

Validation requires experimentally accessible observables that directly reflect mechanical incompatibility. HR-DIC provides strain localization maps and band statistics relevant to claims of strain delocalization [73,130], while diffraction and microdiffraction techniques quantify internal stress partitioning and thereby constrain back-stress evolution and interface constitutive laws. Full-field localization maps, such as those shown in Figure 10, are similarly valuable because they assess the spatial distribution of deformation rather than merely its average magnitude. As such, they provide critical validation targets for models that aim to predict strain banding, hotspot formation, and interlayer strain transfer. In situ neutron diffraction has enabled direct measurement of load sharing in multilayered steels [22], and three-dimensional X-ray microdiffraction has resolved submicron-scale heterogeneity for comparison with microstructure-resolved simulations [28]. Figure 5 illustrates the type of benchmark dataset required for rigorous model validation, as it directly measures internal stress partitioning rather than inferring it from macroscopic response. Such observations are essential for assessing whether a model captures the underlying physics in a physically meaningful manner. In situ microscopy further supports validation of interface models by revealing dislocation pile-up and transmission phenomena (Figure 6) [29].

The absence of reproducible benchmarks remains a major constraint. An effective benchmark should integrate a well-characterized heterostructure with multimodal measurements, clearly specify boundary conditions and representativeness criteria, and provide shared digital microstructure datasets accompanied by metadata. Benchmarks combining stress partitioning, strain-band statistics, and near-interface microstructural characterization would enable meaningful discrimination among continuum, crystal plasticity, and dislocation-based modeling approaches and accelerate identification of missing physics. Reporting standards should therefore include detailed microstructural and interfacial descriptors, boundary condition specifications, mesh resolution relative to heterogeneity length scales, and quantified uncertainties in both microstructural inputs and constitutive parameters.

Benchmarking and data sharing are increasingly feasible given advances in experimental pipelines that generate spatially aligned microstructure and field measurements. Examples include combined diffraction and DIC datasets for multilayered and gradient materials [202,203], as well as integrated EBSD and in situ testing workflows applied to heterogeneous steels and entropy alloys [204,205]. Tomography-informed digital reconstructions have further supported validation of coupled plasticity-fracture simulations [206,207]. For development of interface constitutive laws, benchmarks that report interface character, chemistry, and local slip-transfer metrics are particularly valuable [208,209]. Community datasets containing raw field measurements, boundary condition metadata, and uncertainty estimates enable objective model intercomparison [210,211,212,213,214,215,216,217,218], facilitate development of reduced-order substitutes with controlled error, and strengthen claims of reproducibility. Such practices should be regarded as foundational infrastructure for predictive heterostructure modeling. Publishing calibration scripts, parameter sets, and microstructural metadata alongside manuscripts would substantially enhance reproducibility, while even lightweight repositories containing microstructure statistics and loading histories would reduce duplication of effort across research groups.

## 11. Emerging Directions

**Figure 16:**
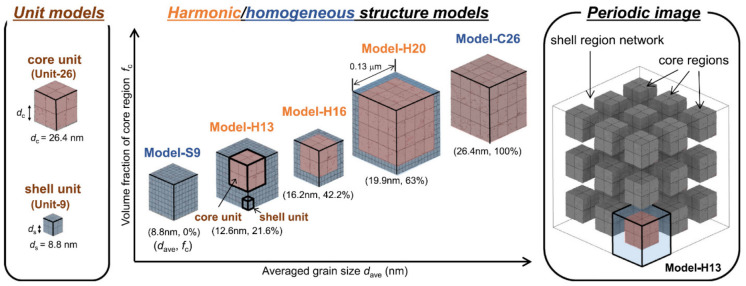
Multiscale modeling concept for harmonic-structure materials combining atomic-scale and dislocation-scale information [177]. The figure highlights a future-oriented strategy for connecting complex topology, defect evolution, and higher-level constitutive response.

Emerging directions in heterostructure modeling are increasingly defined by the reliability with which information can be transferred across length scales and architectural classes as shown in Figure 16. Sequential multiscale coupling remains the prevailing strategy [25,36,37,177]. Within this framework, atomistic simulations bound interface strength and transmission mechanisms; DDD extract statistics of pile-ups and transmission; and crystal plasticity or continuum formulations provide component-scale predictions. A persistent challenge lies in quantifying the uncertainty introduced at each scale transition, particularly for interface populations that exhibit spatial variability within a single specimen [7].

Computational efficiency continues to improve through accelerated solvers and reduced-order representations. Spectral approaches, such as CPFFT, facilitate ensemble analyses for periodic RVEs and rapid evaluation of parameter sensitivities [35,155]. Advances in adaptive strategies and solver technology within CPFEM now permit larger three-dimensional reconstructions and closer integration with experimental datasets. Reduced-order surrogates are most valuable when they preserve the mapping from microstructural descriptors to internal-field observables, such as stress partitioning and strain localization, rather than merely reproducing macroscopic stress–strain curves [25].

Physics-informed, data-driven approaches are also gaining prominence. However, their utility depends on mechanistic interpretability and rigorous verification. Surrogate models that enforce invariance principles, maintain thermodynamic consistency, and explicitly represent the evolution of stress partitioning and localization are more likely to generalize beyond calibration conditions than black-box regressions [7,25]. Concurrently, the use of digital microstructure ensembles and uncertainty-aware prediction is becoming essential, given specimen-to-specimen variability introduced by processing pathways [22,28,219].

Benchmark-driven intercomparison efforts are particularly promising. An effective benchmark should encompass at least one canonical laminate and one canonical gradient architecture, each accompanied by shared diffraction-based stress-partitioning data, high-resolution DIC localization maps, documented boundary conditions, and comprehensive microstructural statistics [22,130]. Open dissemination of digital microstructures, verification cases, and post-processing scripts would reduce duplication of effort and enable transparent, quantitative comparison of reported advances in model fidelity across research groups.

## 12. Conclusions and Outlook

Heterostructured metals achieve strength–ductility synergy because adjacent hetero-zones cannot deform compatibly under applied loading. Subsequent mechanistic analyses attributed the resulting macroscopic strain hardening to internal back and forward stresses generated by this incompatibility. Advances in experimental characterization have rendered these internal fields directly measurable. Layer-scale load sharing has been quantified through in situ neutron diffraction, and submicron-scale mechanical heterogeneity has been resolved using three-dimensional X-ray microdiffraction. Collectively, these studies establish that model credibility depends on accurate prediction of stress partitioning, plastic strain gradients, and the associated storage of GNDs that underpin heterogeneity-induced strengthening and hardening.

Continuum constitutive models remain indispensable for component-scale prediction because they efficiently represent hetero-zones and can be embedded within structural finite element frameworks. Zone-coupled $J_{2}$ plasticity formulations reproduce load sharing when boundary conditions and transition layers are explicitly incorporated. Strain-gradient and kinematic hardening terms introduce intrinsic length scales and internal stress contributions. However, they must be conceptually distinguished to avoid double-counting of GND-mediated effects. Experimental studies of copper–brass laminates have shown that HDI hardening can saturate with increasing strain gradient as dislocation structures reorganize near interfaces. This observation underscores the necessity for back-stress evolution laws to incorporate physically interpretable saturation mechanisms rather than relying solely on numerical damping.

Crystal plasticity provides the minimum crystallographic resolution required to predict texture-sensitive stress partitioning and strain localization. Simulations of gradient TWIP steels incorporating depth-dependent twinning activity have reproduced how gradients alter deformation mechanism sequences and sustain hardening beyond rule-of-mixtures expectations. Dislocation-resolved approaches complement crystal plasticity by clarifying conditions under which pile-ups and transmission statistics govern strength. Three-dimensional DDD simulations have quantified interfacial pile-ups and gradient length-scale effects, while CDD models link GND density to lattice curvature through established kinematic relations. Mesoscale outputs are most impactful when exported as uncertainty-bounded priors for higher-scale models rather than as single deterministic parameter values.

Damage and fracture modeling must treat heterogeneity as an evolving internal field rather than as a static defect distribution. Cohesive-zone and phase-field formulations offer tractable means of coupling plasticity with interfacial decohesion and crack deflection. Crystal-plasticity phase-field simulations have demonstrated how gradients redistribute crack-driving forces and promote deflection rather than penetration. A key next step is to anchor such predictions to experimentally measured evolution of stress partitioning and strain localization, ensuring that failure models remain consistent with the governing deformation physics.

Three strategic priorities emerge for the research community. First, the development of benchmark datasets pairing well-characterized architectures with multimodal internal-field measurements, building on combined diffraction and digital image correlation campaigns. Second, the formulation of interface constitutive laws that remain predictive across processing routes. While atomistic simulations can bound transmission and decohesion strengths, robust upscaling requires systematic sampling of interface populations and propagation of their variability. Third, adoption of uncertainty-aware model comparison and reporting practices. Ensemble-based microstructure representations and probabilistic calibration frameworks are necessary to express predictions with quantified confidence intervals rather than single deterministic curves.

Specialized topics remain underrepresented because they demand either aligned datasets or substantial computation. Examples include interface-specific slip-transfer and decohesion laws constrained by direct observations of slip transmission and dislocation pile-ups, rigorous uncertainty propagation during parameter passing from molecular dynamics to dislocation dynamics and then to crystal plasticity or continuum models, and microstructure-resolved validation campaigns in which digital twins are registered to diffraction and HR-DIC fields rather than only to macroscopic stress–strain curves. These directions are likely to become more central as multimodal, spatially aligned datasets become more widely available.

Some practices are losing importance because they provide limited physical discrimination. Approaches are increasingly difficult to justify when phenomenological gradient or kinematic terms are used mainly as curve-fitting devices, when calibration relies on a single macroscopic stress–strain curve, or when additional internal variables are introduced without identifiability checks and without at least one internal-field validation target. Likewise, purely illustrative simulations based on overly idealized microstructures offer diminishing returns now that experimental reconstructions and ensemble-based representations are feasible.

Fast-moving topics that are shaping current progress include registered digital-twin pipelines that connect three-dimensional microstructure reconstructions to multimodal internal-field measurements, hybrid multiscale workflows that couple atomistics, dislocation dynamics, and crystal plasticity with explicit uncertainty quantification, and scalable surrogate modeling that preserves physically meaningful mappings from microstructure to internal fields. Coupling these developments with benchmarked damage models that track evolving heterogeneity will support a transition from post hoc explanation toward design-oriented prediction in heterostructured metals.

Progress along these lines will enable modeling and simulation to function as reliable design tools for heterostructured materials by linking architecture, internal fields, and failure metrics within validated, uncertainty-aware frameworks.

## Data Availability

No new data were created or analyzed in this study. Data sharing is not applicable to this article.
